# The Bar-On Model and Multifactor Measure of Human Performance: Validation and Application

**DOI:** 10.3389/fpsyg.2022.872360

**Published:** 2022-07-04

**Authors:** Reuven Bar-On, Carina Fiedeldey-Van Dijk

**Affiliations:** ^1^Into Performance ULC, Aurora, ON, Canada; ^2^Department of Psychology, University of Pretoria, Pretoria, South Africa

**Keywords:** Bar-On model, performance, Multifactor Measure of Performance, occupational performance, performance assessment, performance enhancement, whole person

## Abstract

In this article, we describe the ongoing validation and application of the Bar-On model of human performance that is assessed with the Multifactor Measure of Performance (MMP). (The Bar-On Multifactor Measure of Performance (MMP) is the intellectual property of Into Performance ULC.) The MMP is a psychometric instrument designed to study, evaluate and enhance performance. We discuss the meaning and importance of performance, and explain the need for creating and applying a comprehensive model and measure of this construct. To address this need, the MMP is structurally organized to assess and strengthen 18 Core Factors that contribute to performance. Five Ring Factors were added to facilitate a deeper understanding of leadership, industriousness, productiveness, risk for burnout, and coachability. Together, they represent a multifactor approach that focuses on current behavior of the “whole person” by evaluating *physical*, *cognitive*, *personal*, *social*, and *inspirational* factors combined. We discuss the properties of the MMP’s normative population, as the baseline for accurate reporting, tailored to different workplace activities and needs. Possible limitations of the research are indicated, together with the need for additional studies to address them. We reflect on the MMP within the Unified Validity Framework and conclude with recommendations for researchers and practitioners to apply this model and measure.

## Introduction

In this section, we discuss the meaning and importance of performance. As a construct that is both fundamental to and omnipresent during the human lifespan, performance needs to be clearly anchored in a comprehensive model. This led to creating the Bar-On operational definition of performance and a scientific means to accurately assess it with the Multifactor Measure of Performance (MMP). Throughout the article, we refer to the fourth and current version of the MMP that we co-developed. Previous versions of the MMP, and the initial research involved in developing and validating them, were published by Bar-On (2016, 2018), Conroy (2017), and Murphy (2018). These revisions of the MMP, made over a period of eight years, have created a scientifically constructed, normed and validated assessment.

Since psychology emerged as a field of experimental study in the mid-nineteenth century, virtually all psychological constructs developed and studied are associated with performance (Murray and Link, 2021). For example, developmental psychology is concerned with physiological, cognitive, personal, social as well as motivational activity and functioning throughout the human lifespan (Britannica, 2017). Pioneers like Piaget, Erikson and Skinner demonstrated that human beings learn to perform, from early development onward, to achieve important goals such as learning how to walk, talk, be more independent, as well as how to perform interpersonally. Educational performance in school is associated with learning new tasks, recalling what is important and useful, and solving problems. Occupational performance involves leaning how to acquire efficient work habits, strategies and expertise to apply throughout one’s career (Ford and Leist, 2021).

Succeeding in life depends on how well individuals perform, and performance is vital to survive and thrive. High performing employees contribute to organizational performance, productivity and profitability. To understand the essence of this concept, we describe the term *performance* as action taken for the purpose of achieving some desired outcome or goal and to bring about a change for the better. This definition is essentially the axiomatic foundation of the present article.

Considering the importance of performance throughout life, it is critical to scientifically and accurately define, assess and enhance this construct. Such an endeavor is also a timely pursuit for employees, leaders and organizations in today’s changing work environment. Problems with performance appraisals are commonly noted and while performance is pervasive, accurate measurement of performance has been elusive to date. The MMP appears to be the first psychometrically validated measure of performance. Bar-On (2016) argued that a performance model and measure of the “whole person” should generate more comprehensive and useful results than performance appraisals, assessment centers, and other existing measures typically provide. His review of existing approaches aimed at understanding and improving performance indicated a critical need for a model and assessment to concomitantly evaluate multiple key contributors to performance (Bar-On, 2018). He reasoned that this would also reduce the need for administering multiple, time-consuming and costly assessments.

Bar-On (2016, p. 104) sought to more accurately understand “why some people perform better than others.” He purposely attempted to be atheoretical in reviewing existing definitions of performance and well-being (Bar-On, 1988). This approach enabled the examination and potential inclusion of a wide array of contributors to performance from an emic-etic perspective. It culminated in his operational definition and conceptual model of human performance.

According to the Bar-On model, an individual’s current level of performance can be explained by the combined strengths and balance of *physical*, *cognitive*, *personal*, *social*, and *inspirational* factors. These five key factor categories can be seen as metaphorically functioning in a sphere. They comprise 18 Core Factors that significantly contribute to performance. When they contribute meaningfully to facilitate competent functioning, the individual is expected to perform well. If one or more factors are significantly challenged, the other factors inside this sphere compensate and support continued performance, however possible.

Using data generated by previous MMP versions, we empirically and conceptually refined the Bar-On model of performance, corroborating its structure, reliability and validity. By design and description, it is not an assessment of personality that evaluates the disposition to behave in a certain manner (Cattell, 1946), neither a measure of cognitive intelligence that estimates the ability to perform based on IQ level (Wechsler, 1958), nor a test of emotional intelligence that is assessed by EQ (Bar-On, 1997). More precisely, the MMP does not assess the potential to perform but rather performance itself based on current behavior.

## Materials and Methods

In this section, we describe key characteristics of the current version of the MMP associated with its validation and application. We explain scale and item refinement, the development of an advanced response scale, and the scoring algorithm construction designed to accurately report current performance. We briefly describe how data were collected and balanced for norming the MMP, and how its psychometric properties and strengths were examined. Last, the methods used to validate this measure are described from different perspectives.

### Scale and Item Refinement of the MMP

Bar-On (2016, 2018) initially identified factors in the international literature thought to contribute to performance. Additionally, he asked 67 individuals from different countries who published research on performance to describe this construct and what impacts it. This expert input helped confirm as well as potentially expand his selection and description of contributors to performance. After creating a large item pool based on these descriptions, the psychometrically strongest items and scales were retained from three earlier MMP versions, reducing the scales from 28 to 23, and the items from 216 to 142.

From 2019 to 2021, we continued refining the MMP to create the current version of this assessment. An iterative process of progressive elimination resulted in a set of 120 items loading on 18 core scales and 5 ring scales, as well as adding two reliability and validity indices, and one additional scale for personalized benchmarking (Fiedeldey-Van Dijk and Bar-On, 2021). We retained 107 of the previous 142 items and added 13 new items, primarily for developing the ring scales.

The ring scales were created by conceptually grouping core factor items thought to contribute to leadership, industriousness, productiveness, risk for burnout, and coachability. This was followed by examining descriptive parameters, item-scale correlations, and internal consistency, to select the strongest items in each of these groupings and explore their factorial structure. Validation analysis is ongoing to evaluate their factorial and predictive strength.

### Development of an Advanced Response Scale for the MMP

After the 5-point Likert and percentage scales confirmed psychometric shortcomings in earlier MMP versions (Bishop and Herron, 2015; Bar-On, 2016, 2018; Fiedeldey-Van Dijk, 2019a), an advanced response scale was created. Self-rated responses tend to be negatively skewed as individuals frequently provide responses on the higher end of response scales (Dolnicar et al., 2011). Despite their popular use in assessments, this typical response style restricts the range within which responses vary, negatively impacts the accurate interpretation of results, and decreases test validity. Respondents have frequently suggested that they feel comfortable with more than five response options, and this paved the way for a new response scale with expanded descriptions.

After careful consideration, we addressed the above-mentioned response challenges with the following format for the current version of the MMP: *Never* (1), *Almost Never* (2), *Very Infrequently* (3), *Infrequently* (4), *Sometimes* (5), *Frequently* (6), *Very Frequently* (7), *Almost Always* (8), *Always* (9).

This format is, by design, positioned at the interval level of scoring. Each point along the scale is numbered to resemble equal distances as well as textually described in symmetrical gradations as recommended by Likert (1932). It allows for sufficient response expression of current behavior, i.e., orienting the response format towards the factors being assessed (Linacre, 2002). It also discourages a tendency to simplify the scale with clearly distinguishable response options. This format is easy to use on mobile devises and touch screen interfaces, which is an important feature for online testing today (Fryer and Nakao, 2020).

Ideally, all scaled response options should be used (Davis and Boone, 2021). We carefully worded items for neutrality within the performance scale they are allocated to, which helped increase the gradual distribution of item responses around the fifth option as the scale midpoint. This effectively reduces skew and elicits responses that are approximately normally distributed (Gorsuch, 1997). To prevent what Linacre (2002) calls “dead zones” in responding, we purposely used a nuanced description of every pair of uneven-even numbered response options. This retains simplicity and facilitates continuation in the meaning of the symmetrical response options. The design also helps minimize occurrences of floor and ceiling effects, and enhances precision in measurement.

The Rasch rating scale analysis offers a way to improve the utility of MMP performance factors, which is planned for future emic-etic data analysis. In preparation, we used its basic principles to rationalize how current response style patterns in the MMP’s normative population might meet the relevant guidelines proposed by Linacre (2002). Prior to removal of outlier, systematic, or indiscriminate responding in the MMP sample (*n* = 4,193), item responses were reasonably distributed between 0.83 and 21.61% across the nine response options. They increased gradually, and unimodally pivoted around the seventh response option for the factors being assessed, which conformed to expectations about self-reports and on a construct that some might view as contentious. These patterns were the incentive for creating the MMP’s current scoring method described below.

### Scoring Algorithm of the MMP

Despite the benefits of including an advanced nine-point response scale, the MMP remains a self-report with all the challenges of response bias that still need to be addressed. Fundamentally, response bias can affect the factor structure of an assessment (Salas-Blas et al., 2022). To address these problematic issues, Fiedeldey-Van Dijk (2019c,2020) created a sophisticated and novel scoring mechanism for the MMP to mitigate the negative impact of response patterns encountered in self-reports. It comprises five methodological concepts, described below, that are based on the individual’s self-image (SI), which frequently affects responding to assessments and challenges the interpretation of results.

#### SIA – Accuracy

When responding to test items, what one individual sees as low could be viewed as high by another. SIA addresses potential response inaccuracies resulting from different possible interpretations of the response-scale format. Personal styles affecting the responses provided are statistically converted to *z* scores to help neutralize their impact, which enhances the accuracy in interpreting scale scores.

#### SIB – Bias

Many individuals will maintain that they perform well. SIB addresses the tendency of individuals to respond in a way that they think will be interpreted as performing well, which is often driven by their predispositions to and assumptions of what is being evaluated and of themselves. This, in turn, creates negative skewness in self-rated responses and challenges scoring accuracy. This type of response bias is addressed by a mathematical cube-root transformation of the *z*-scored responses to items.

#### SIC – Consistency

How individuals respond can depend on the immediate situation and circumstances. SIC addresses a possible pattern of inconsistent responding that affects scale scores, which results in unreliable interpretation. When a set upper threshold is exceeded, it is addressed individually rather than systemically as part of scale scoring.

#### SID – Desirability

Some individuals attempt to simulate that which they want to portray about themselves. SID addresses the tendency of individuals to present themselves in a certain manner for various reasons. This can bring into question the credibility of the individual’s results. Similar to SIC, when either a set upper or lower threshold is exceeded, one can explore the reasons for this when debriefing the results and/or during coaching.

#### SIE – Equality

Inherently, individuals compare themselves to others for one reason or another. SIE applies score standardization to provide a fair baseline for viewing assessment results. This is widely used to address natural and expected fluctuations in scale and individual scores. The standardization process converts raw scale scores to T scores (mean = 50; standard deviation = 10) for all MMP scales.

Whereas SIA and SIB address systemic response processes at the item level, SIE is applied at the scale level to facilitate more meaningful interpretations of scale scores. Scale score interpretation is further enhanced by considering SIC and SID scores as described above.

### Data Collection and Norming of the MMP

The use of online psychometric assessment is growing globally *via* different types of electronic devices. This practice increases reach and provides real-time results about employee performance that can assist in hiring and development decisions across the career span (cut-e Group, 2016). Bar-On collected the MMP responses of 4,193 individuals from 2014 to 2019 using non-probability sampling by making the assessment accessible online, and by reaching out to small-, medium- and large-sized organizations. More than 80% of US Fortune 500 and 75% of United Kingdom Times Top 100 companies use psychometric assessments (Dua Dullu, 2017).

All respondents completed the assessment online, which facilitated expansion of a broad geographical sample representation from North America into Europe and beyond. This effort created a sufficiently large pilot sample to overcome the limitations of non-randomness. All MMP items contained non-missing responses, and we subjected the sample to two initial processes, namely data *verification* and *balancing*. They contributed to low standard error of mean scale scores as a reliable indicator of sample representation (Barde and Prajakt, 2012), and helped regulate the effect of demographic covariates in factoring and prediction, as shown in Section “Results.”

For data verification, we addressed common issues known to compromise validity. This included the following extreme criteria to delete cases with abnormal response patterns across all MMP item responses: An outlying high mean; large mean-median-mode differences; small or large standard deviation (SD); inter-quartile or response range; skewness and leptokurtosis; and/or a low degree of self-rated openness, accuracy and honesty in responding. We identified an average presence of 1.22 (SD=1.46) for extreme criteria per case, with zero extreme criteria among 40.01% of cases. We excluded 362 respondents (8.63% of the sample) from MMP validation based on cases that obtained more than three extreme criteria. This created a verified sample size of 3,831.

For sample balancing, we sought wide demographic proportionality with sensitivity toward Human Rights compliance. Variables that meaningfully segmented participants by demographic character were included to support validation research and generate specialized norms for scoring. We kept these variables contained to keep response time brief and minimize drop-out. Older males working in public safety were over-represented and therefore reduced through randomization followed by a systematic selection of odd or even numbers from these demographics. The resulting balanced sample (*n* = 3,039) effectively represents the normative population for MMP validation and scale-score standardization, distinguished by its composite character with performance scores across the continuum.

The normative population is 54.89% male and 45.11% female. On the average, these adults are 40.30 years of age, categorized as young (<30, 22.89%), young to middle-aged (30-39, 27.01%), middle-aged (40-49, 23.51%), older (50-59, 19.61%), and retirees or near retirees (>59, 6.97%). The highest educational qualification they obtained comprises the following: Not yet completed high school (4.10%); high school completion with a diploma or certificate (31.30%); a Bachelor’s degree (40.04%); Master’s degree (23.26%); and Doctoral degree or equivalent completed (1.30%). They also represent 104 different listed occupations.

Individuals included in the normative population are 71.77% North American citizens, while the remainder represent 75 different countries, primarily from the United Kingdom, Ireland, Scotland, Australia, and other English-speaking countries. Despite the diversity of countries, dominance of spoken English as opposed to other first-languages classifies the current normative population as emic (Lindridge, 2015). Our current secondary focus of cross-cultural inclusion started a process of etic-strengthening far beyond the current 28.23% in the MMP normative population and of researching its impact.

Additionally, the normative population consists of 33.66% individuals with no managerial responsibility versus 25.82, 20.36, 11.31, and 8.85% with first-line, mid-level, senior, or top executive managerial positions, respectively. It also includes marginal, average and top performers, based on their recent performance ratings, with broad levels of reported risk, stress and job satisfaction associated with their work. The diverse character of the normative population forms the premise for different representative norm options that the MMP offers in assessing performance.

Data collection took place outside of academic, medical, and government settings. The MMP introduction clearly conveyed that participation indicated voluntary consent and that participants would contribute to research as an aggregate. The participants were anonymous and individual results were kept confidential to comply with standard Institutional Review Board (IRB) requirements.

### Statistical Rigor Behind the Bar-On Model and MMP

We conducted descriptive, inferential and multivariate statistical analysis with SAS software, using the MMP normative population, as well as samples provided by Conroy (2017) and Murphy (2018). The psychometric properties of MMP items and scales were examined with descriptive statistics (arithmetic mean, SD, minimum and maximum values, standard error of the mean, skewness, and kurtosis) to reveal performance patterns and relationships within the responses of the normative population. Linear relationships were determined between items and scales using the Pearson’s Product-Moment correlation coefficient.

Chi-square contingency associations were used to describe demographic heterogeneity in the balanced normative population. We tested multiple subgroup differences, accommodating for unequal sizes using general linear modeling (specifically Analysis of Variance) with Sheffé’s post hoc analysis, or used Student *t*-tests for examining two subgroups for differences. These statistics helped determine possible test bias and verify test fairness, suggesting what might underlie resulting performance levels, subject to sample size and randomness (Ferguson, 2009).

The practical significance of the magnitude of mean difference in factor performance between subgroups was examined with Cohen’s *d*. Overestimation of differences when using 0.20, 0.50 and 0.80 as small, medium and large effect size for interpretation, is well documented (Paterson et al., 2016; Merino-Soto and Copez-Lonzoy, 2018). We thus lowered minimum guidelines to 0.15, 0.36 and 0.65 respectively, following Lovakov and Agadullina’s (2021) recommended use in broad social-psychology contexts. We approached effect size conservatively, since performance as a construct carries substantive significance with notable business consequences in specific fields of work (Kelley and Preacher, 2012). The same techniques were applied in two external studies to examine performance differences between two distinct groups (Conroy, 2017; Murphy, 2018).

In prior versions of the MMP, Principal Components Analysis (PCA) was preliminarily carried out on the raw responses of experimental item pools as obtained using different response scale formats, in support of early item and scale development. In the current version, we paused data collection and sought to find a strong factorial structure and a-priori model specification for performance that is easy to understand and makes good theoretical sense (Kline, 2002; Hill and Lewicki, 2006). Upon reflection, we achieved a substantial sample size, with developing sample representation. We expect some measurement error will exist based on the adjustments that were made in the wording of some items and in the response-scale format. Hence, we intentionally refrained from hypothesizing about the number of factors and from anticipating specific factor loadings (Gorsuch, 1997). Our preparation lays the groundwork for focusing efforts to obtain confirmation of factor consistency in future studies.

After SIA and SIB application, the *z*-score cube-root responses based on a narrower and more refined item pool were subjected to PCA to examine the factorial structure of the MMP. PCA is a linear dimensionality reduction technique that converts the set of correlated behavioral items in the high dimensional space of performance into a series of uncorrelated components in the low dimensional space of condensed components. While both PCA and Principal Axis Factoring (PAF) are reduction techniques (Mabel and Olayemi, 2020), we purposely positioned the comprehensive array of different performance factors as outcomes of current behavior composites that can change and be further developed. Hence, we applied PCA rather than PAF, where performance factors are treated as latent factors that affect current behaviors. In the PCA approach, these uncorrelated performance factors are also called principal components.

We limited the structure iteratively from 14 to 22 output factors to arrive at the conceptually and statistically clearest factorial structure possible through reduced dimensionality, as well as strong inter-individual variance, and achieve face equivalence. Despite expected measurement error, minimalized overlap in factors was evident in the initial principal components – a factor method of linear item combinations – and, hence, we expected cross-loadings to be largely absent. However, we sought to produce a simple and replicable structure of principal components that would contribute to performance, which led to the decision to apply rotation to the data.

Orthogonal rotation methods enhance the distinction between components that account for the overall construct, in this case performance, and simplify their interpretation for different applications. The specific method selected can significantly impact the magnitude of item cross-loadings and inter-factor correlations with implications for construct validity, dimensionality, and ultimate scale scoring (Sass and Schmitt, 2010). Optimization criteria differ somewhat between rotation methods, but rotation solutions for uncorrelated factors are reasoned to produce similar model fit (Brown, 2009).

We applied Varimax rotation to maximize the variance of the factors and facilitate interpretation of its dimensionality (Hill and Lewicki, 2006). This commonly applied orthogonal method uses a mathematical algorithm that maximizes high- and low-value factor loadings, and minimizes mid-value factor loadings. The first principal factor, which accounts for most of the variance, tends to diminish due to the redistribution of factor variance (Rennie, 1997). This effect complimented how Bar-On conceptualized the model as multi-factored from the outset. Varimax rotation minimizes the complexity of a factor *via* its number of items, which is achieved through balancing this reciprocal relationship (Sass and Schmitt, 2010). We adopted Timmerman and Lorenzo-Seva’s (2011) suggestion to substantiate the construct dimensionality as revealed by PCA with conceptual considerations as well.

Our three-step use of PCA, Varimax rotation, and the Kaiser-Guttman Eigenvalue Criterion is known as the Little Jiffy, Mark IV routine (Kaiser and Rice, 1974). Since this popular sequence is subject to criticism (Gorsuch, 1997), we applied additional precautions. For example, in selecting the number of factors to retain, we included the Scree test (Kaiser’s criterion) and applied Eigenvalues >1 conservatively in the interest of parsimony, culminating at >1.2. This helped avoid unwanted error variance and possible over-factoring, even when this is preferred to under-factoring (Gorsuch, 1997). Even though the PCA was based on a large and heterogeneous sample, which helped minimize attenuation of correlation coefficients between the resulting factors (Golino and Epskamp, 2017), we verified that they were low (>0.3) to moderate.

Another precaution was that we avoided the selection of factors with complex loadings, i.e., where two or more concepts can collapse under one factor pointing to under-factoring (Fabrigar and Wegener, 2012; Bandalos and Gerstner, 2016). Simplicity in the factor structure, combined with the size and character of the sample, enabled adherence to two criteria: A minimum of three items per factor, and ideally more; and a minimum factor loading of 0.30 to 0.40 for item inclusion in the proposed structure in the absence of cross-loadings (Cattell, 1970; Tabachnick and Fidell, 2001; Yong and Pearce, 2013). We followed-up by identifying any item that single-loaded below a salient 0.40 on a factor, and using both its conceptual contribution and distributional properties to help refine item wording. These actions reduce the possibility of finding false negatives with factor structure replication and are expected to enhance precision in future studies.

Multiple steps were applied to this version of the MMP, which led to key improvements for validation and application. Regardless, it appeared evident, from its earliest development, that its structure would be multi-factored. This is favorable as an indication of measurement invariance from preliminary versions to the current version of the MMP, and across various demographic groups as demonstrated in different samples (Putnick and Bornstein, 2016). The emerging pattern suggests early stability in and psychometric equivalence of the Bar-On model of human performance. Small refinements in a number of items, where appropriate, ruled out the benefit of running supplementary methods to determine the number of factors at this junction, such as parallel analysis or by separating factoring in sample halves. However, these methods are recommended for added rigor in the future.

It is important to consider whether MMP scales measure systematically, i.e., whether they are sensitive towards demographic differences as a result of differential item functioning rather than actual subgroup differences. Factor equivalence across demographic groups produces precise and fair performance measurement, in the absence of which, test bias can occur (Bauer, 2017). Measurement invariance will be examined when the growing normative population reaches etic representation, and when covariances among demographic characteristics in the sample are further reduced by expanding and strengthening sample representation. This will be suitable with item-response theory (IRT) or structural equation modeling (SEM), and more specifically with plans to conduct Confirmatory Factor Analysis (CFA).

Two considerations might moderate the presently unknown possibility of measurement invariance, and justify that comparison of performance results between demographic groups could be meaningful. First, the large size of the MMP normative population reduces the power and sensitivity to detect differences in absolute model fit based on Chi-square as the sole criterion to evaluate it, and possibly lead to over-rejection of measurement invariance. Second, since the number of MMP items and factors are large and the number of subgroups that are compared can be substantially more than three, Putnick and Bornstein (2016) suggested that cut-off thresholds for testing measurement invariance might need to be relaxed or perhaps corrected in some cases.

Internal scale consistency was computed with Cronbach’s coefficient alpha, and item-scale correlations were examined for an intended range between 0.30 and 0.60 (Tabachnick and Fidell, 2001). Cronbach alpha is a lower-bound reliability measure that estimates the proportion of the variances in all items accounted for per MMP scale. It is grounded in tau-equivalence, which assumes that each item measures the same latent trait within their allocated scale (Tavakol and Dennick, 2011). The application of a fixed Cronbach alpha threshold becomes somewhat arbitrary, the more this assumption becomes violated. When the number of items in each scale is small, they do not fully cover the breadth of the scale concept, and their standard deviations are not equivalent, reliability might be underestimated. On the other hand, scales containing redundant items will overestimate reliability estimates. We considered the application of the restrictive tau-equivalent model acceptable and meaningful for the sake of parsimony (Graham, 2006).

Alpha scores above 0.70 are generally sought for unidimensional factors (Anastasi, 1988; Vogt and Johnson, 2011), which allows for a 0.51 error variance in scale scores. By comparison, a threshold set at 0.80 decreases the fraction of factor scores that is attributable to random error to 0.36. Understated conditions could render a score of 0.70 acceptable. To address this point, MMP items and scales are based on response conversions to cube-root *z*-scores, which renders a more uniform response distribution with a slightly decreased variation. This situation results in moderate and acceptable reliability, while underscoring scale independence. In the absence of this scoring process, possible inflation effects are often embedded in reported alphas, especially when based on self-ratings that are prone to unconstrained negative skews.

We conducted forward stepwise Multiple Regression Analysis to explore the best predictive equations containing a specific combination of MMP core scales that explain the variability in a dependent (criterion) variable (Vogt and Johnson, 2011). The 18 core scales were examined against each of the five ring scales, and against transformational, transactional and passive-avoidant leadership styles.

The large size of the normative population justified the use of a conservatively set and supple threshold for statistical significance as *p*<0.01 when differences were generally sought, and *p*<0.05 when not, to maintain high standards for MMP validation (Chawla, 2017; Vyse, 2017; Wasserstein et al., 2019).

## Results

In this section, we summarize and conclude that the empirical findings from the MMP’s normative population adequately demonstrates that it is a valid, reliable and applicable psychological assessment anchored in the performance model on which it is based. We also discuss the scale structure of the current MMP version. This includes the validity and reliability indices that are designed to evaluate and increase response integrity, as well as the perceived current level of performance. We describe key characteristics of the MMP associated with its validation and application. We close with a description of its practical characteristics and features suitable for widespread application.

### Factorial Structure of the MMP

The Bar-On model and measure of human performance comprises a conceptually well-defined 18-factor structure supported by Principal Components Analysis. PCA was applied to 101 items. Eigenvalues ranged from 7.04 to 1.27 for 19 principal components with minimal risk for under-factoring, with a steady and natural drop towards the downward curve in the Scree plot (Fiedeldey-Van Dijk and Bar-On, 2021). The PCA results are summarized in Table 1, which reveals that 18 factors met our described criterion requirements and empirically demonstrated the best conceptual fit for a distinct MMP structure. The variance explained by each principal component ranged from 3.96 to 1.64%, satisfactorily explaining 47.52% of performance as described by the Bar-On model.

**TABLE 1:** Summary of PCA results in support of the MMP 18-factor structure (*n* = 3,039).

Principal component number	Variance explained by principal component	Number of items in principal component	Principal component loading		Evident MMP scale supported by principal component
			Highest	Lowest	
1	3.96%	11	0.62	0.37	Ingenuity
2	3.40%	9	0.72	0.45	Perseverance
3	3.12%	8	0.69	0.42	Self-Control
4	3.10%	6	0.82	0.35	Wellness
5	2.85%	6	0.76	0.53	Engagement
6	2.77%	7	0.72	0.49	Applying Experience
7	2.54%	5	0.76	0.56	Self-Reliance
8	2.51%	5	0.72	0.39	Connectedness
9	2.47%	5	0.76	0.36	Social Awareness
10	2.46%	5	0.70	0.35	Courage
11	2.40%	6	0.66	0.41	Finding Meaning
12	2.29%	4	0.54	0.50	Coping
13	2.28%	4	0.74	0.63	Discomfort Tolerance
14	2.21%	6	0.58	0.41	Decisiveness
15	2.06%	5	0.67	0.31	Problem Solving
16	1.99%	3	0.75	0.66	Protectiveness
17	1.80%	3	0.67	0.62	Self-Understanding
18	1.67%	0	–	–	–
19	1.64%	3	0.58	0.31	Motivation

*Principal components extraction method; varimax orthogonal rotation method; 101 MMP3 items.*

In meeting a recommended target of 60% of explained variance (Hair et al., 2018), the last six of 32 MMP factors eventually dropped to an Eigenvalue of 0.88. This falls below the recommended minimum of 1.00 to meaningfully account for variance, and produces small factors that have weak underlying psychometric support with little interpretational value. Instead, by applying a minimum acceptable target of 50% of explained variance (Streiner, 1994), with all Eigenvalues above 1.00, 22 principal components in MMP item responses qualified for consideration. However, principal component extractions greater than 19 failed to meet the inclusion criterion of a minimum of three items per factor, and weakened the strength of some factor structures within the overall factor pattern. Therefore, we considered the explained variance based on 18 discernible principal components acceptable according to Streiner’s recommended target, and justifiable on theoretical and conceptual grounds as well.

Communality estimates between items, which indicate how much variance for each principal component can be created through factor extraction, were stable. Factor loadings primarily ranged from 0.33 to 0.70 with many approaching 0.60 as targeted. Root mean square off-diagonal residuals were low at 0.03 overall and below 0.10, which supports the argument that no additional components could have been extracted and that an 18-factor MMP structure is not in violation of parsimony. No factor loadings were smaller than 0.30 (Kline, 2002), and the weakest item among 11 factors achieved salient loadings larger than 0.40, while only two items might contain some inter-item dependency with factor loadings of 0.80 and 0.82 (see the Supplementary Material for details). Because of this, we minimally refined the wording of items outside of the 0.40 to 0.80 factor-loading range to retain them for their conceptual value and contribution to a single principal component. Overall, the factor loadings supported the MMP’s structural validity of 18 core factors. We used the item factor loadings to describe each MMP factor in a precise manner.

### Core Factors Assessed With the MMP

The 18 Core Factors, as grouped into five factor categories, are described below.

#### Physical Factors

• *Wellness* describes how physically fit and well individuals feel. This evaluates how they feel about their physical appearance as well as eating and sleeping habits, and the degree to which they feel refreshed in the morning and energetic during the day.
• *Discomfort Tolerance* reveals how willing individuals are to endure working long hours, sleeping less and eating at irregular times to complete work on time and meet deadlines.

#### Cognitive Factors

• *Problem-Solving* evaluates how effective individuals are in addressing challenges by attempting to logically understand them and arrive at ways to methodically deal with them. This requires collecting relevant information, weighing conflicting evidence and ambiguity, as well as considering the short- and long-term consequences of potential solutions.
• *Applying Experience* indicates how efficient individuals are in relying on familiar or proven methods to address current challenges, building on successes and avoiding repeating past failures. This is about using experience in deciding on what is and is not worth applying.
• *Ingenuity* reveals how flexible and resourceful individuals are in making decisions when situations are unexpected and unpredictable or become complicated. This also indicates how resilient they are when matters do not turn out as planned, by improvising, making needed adjustments to overcome challenges, and adapting to change.

#### Personal Factors

• *Self-Understanding* evaluates how well individuals know who they are, why they behave in certain ways, and why they feel the way they do. This indicates how effectively they look inward and engage in self-reflection, which leads to enhancing self-insight.
• *Self-Reliance* evaluates how self-sufficient individuals are by depending on themselves more than on others for help in making decisions. This is about acting independently, while being open to receiving input from others.
• *Self-Control* describes how effective individuals are in managing their emotions and impulses, so they are not disruptive in their relationships with others. This includes exercising restraint and retaining self-composure in anxiety-provoking situations.
• *Coping* is about how efficiently individuals handle pressure and stress. This reveals how well they understand these challenging situations and then function effectively in dealing with them.
• *Decisiveness* indicates how assertively individuals deal with situations. This includes how they express themselves confidently and act boldly when necessary, without being aggressive or hostile.
• *Courage* evaluates how successful individuals are in handling their apprehension and fear to function effectively in potentially dangerous and even life-threatening situations. This includes preventing others from being physically harmed, which is grounded in a clear sense of moral responsibility.

#### Social Factors

• *Social-Awareness* describes how alert individuals are to understanding what others need, feel and communicate both verbally and non-verbally, which facilitates interacting with them.
• *Connectedness* evaluates how successful individuals are in establishing and maintaining relationships with others. This includes getting along with family, friends and colleagues, as well as how well they enjoy social interaction in general.
• *Protectiveness* indicates how willing individuals are to support and defend others who are treated unfairly, irrespective of potentially negative consequences for themselves. This includes being sympathetic toward others, which is anchored in having, expressing, and living by a clear set of social values.

#### Inspirational Factors

• *Finding Meaning* evaluates how actively individuals pursue living a more meaningful life, which has a positive impact on others as well as on themselves. This describes the process or journey that leads them to achieve a sense of meaningfulness in their work and elsewhere, helping define who they are and what they do as individuals.
• *Engagement* indicates how energized individuals are and how positive they feel about their work, involvements and accomplishments, which stimulates them to do and contribute more.
• *Motivation* evaluates how excited and driven individuals are about what they are involved in, and what they want to do in the future. This also includes how effectively they focus on elements that bring them enjoyment in their work and elsewhere.
• *Perseverance* reveals how determined, committed and persistent individuals are in following through with decisions that are made and in achieving goals.

It is important to note that the six Personal Factors, described above, lend themselves to a potential split orientation. Its structural merit will be statistically re-examined with that of the other factor categories based on representative samples going forward. The first three factors in this category appear to share a more internal and self-oriented characteristic, while the last three appear to be more external and self-other oriented. These apparent differences are useful in debriefing MMP results to gain more insight into how they impact performance. The distinction is also helpful in coaching to strengthen them for individuals to perform on a higher level.

### Ring Factors Assessed With the MMP

While the 18 Core Factors reveal how well individuals are currently performing overall, they are metaphorically surrounded by 5 Ring Factors that indicate how performance is displayed at work. These factors are described below.

• *Leadership* describes how successfully individuals stay on target and make good choices. It is about how well they deal with difficulties under pressure, especially during times of uncertainty and stress. This is also about how confidently they take decisive action, and modify decisions when needed.
• *Industriousness* shows the importance that individuals place on working hard to achieve results. It has to do with the degree of commitment and proficiency that they display. This is also about how efficient they are in planning, time management, and in applying technology.
• *Productiveness* indicates the emphasis individuals place on working smart to tactically deliver results. It has to do with how well they work strategically and maintain perspective. This is about how they actively seek opportunities and effectively monitor the value of their accomplishments.
• *Risk for Burnout* reveals how close individuals might be to becoming excessively exhausted. It suggests that they might have been pushing themselves too hard over an extended period of time at work or elsewhere.
• *Coachability* suggests how readily and meaningfully individuals are expected to respond to efforts designed to enhance their performance. It implies how well they might benefit from investing in coaching, mentoring or group training for the purpose of further self-development.

### Additional Metrics of the MMP

Three other scales were included to help interpret the MMP results.

#### The Reliability and Validity Indices Designed to Examine Response Integrity

Two indices evaluate MMP response integrity, namely *Self-Image Consistency* (SIC) and *Self-Image Desirability* (SID).

SIC is a reliability index that indicates whether individuals provided similar responses to closely related MMP items. Low SIC scores could indicate possible random responding, or might suggest that their level of self-understanding is still maturing and/or not yet fully developed. By comparison, SID is a validity index that estimates how accurately they describe themselves when completing the MMP. High SID scores suggest that they might have over-rated their adeptness and strength in the Core Factors assessed, while low SID scores could indicate that they were overly self-critical in responding to the assessment.

Acceptable SIC and SID levels were determined in the normative population, indicating reliable response integrity (Fiedeldey-Van Dijk, 2019b). Since we found no empirical grounds for adjusting scale scores as part of the scoring algorithm, we recommend that SIC and/or SID scores falling outside of the acceptable range should be dealt with on a case-by-case basis. For example, understanding the respondent’s particular circumstances might adequately explain high or low SIC and SID scores. If the MMP results still remain questionable and could possibly be invalid, they should probably be excluded from a group profile and report. The individual should also be asked to retake the assessment as openly and honestly as possible.

SIC and SID occur sporadically in MMP responding. Therefore, they are addressed individually when debriefing the MMP results, or when deciding whether to include the profile in an MMP report or group profile. By comparison, SIA and SIB address systemic response processes at the item level, while SIE is applied at the scale level to facilitate more meaningful interpretations of scale scores.

#### The Perceived Current Level of Performance

Individuals frequently approach assessments with a preconceived notion of how they are or present themselves to others regarding the construct being assessed, which could be realistic, idealistic, over-rated or under-rated (Paulhus, 1984). While this perception will naturally influence how they respond, it can also offer valuable insights when interpreting the results with the MMP’s *Current Level of Performance* (CLP) scale. This metric indicates how individuals view, or wish to view, their present performance and convey this to others. When in reasonable alignment with other MMP scale scores, CLP serves as a personalized benchmark that can help interpret their results.

CLP should not be equated with one’s average level of performance, which would run the risk of over-simplifying, misleading and/or masking the nuances of individual contributors to performance. Performance is best evaluated and understood by examining the combination, balance and strength of the key factors that contribute to it.

### Psychometric Properties of the MMP Scales

Strength in psychometric properties of MMP items and scales is critical for the assessment’s reliability and validity. The results in Table 2, which are based on cube-root *z* scores, list scale means that should lie close to 0 (except for SIC and SID). Individuals rated themselves comparatively lower in Wellness, Self-Reliance, and Protectiveness, and higher in Finding Meaning and Applying Experience. After score standardization (based on applying SIE to obtain T scores for each scale) however, scale scores can be compared directly with each other for interpretation as they share the same baseline.

**TABLE 2:** Psychometric properties of the MMP scales (*n* = 3,039).

Factor/ Scale	Mean	SD	Min	Max	Standard error of mean	Skewness	Kurtosis
WL	−0.20	0.56	−1.35	1.13	0.0102	0.12	−0.97
DT	0.20	0.66	−1.40	1.33	0.0120	−0.52	−0.79
PS	0.26	0.46	−1.14	1.09	0.0083	−0.56	−0.49
AE	0.47	0.43	−1.21	1.20	0.0078	−1.01	0.50
IG	0.25	0.43	−0.97	1.09	0.0079	−0.47	−0.67
SU	0.19	0.52	−1.22	1.22	0.0094	−0.43	−0.65
SR	−0.10	0.61	−1.39	1.40	0.0110	0.13	−0.90
SC	0.02	0.54	−1.26	1.12	0.0099	−0.24	−0.94
CP	0.15	0.47	−1.18	1.06	0.0086	−0.43	−0.64
DC	0.03	0.45	−1.17	1.04	0.0081	−0.23	−0.57
CR	0.15	0.60	−1.27	1.22	0.0108	−0.29	−1.04
SA	0.01	0.58	−1.27	1.20	0.0106	−0.02	−0.95
CN	0.23	0.53	−1.22	1.20	0.0096	−0.52	−0.54
PT	−0.02	0.61	−1.41	1.21	0.0111	−0.05	–0.98
FM	0.49	0.42	−1.14	1.25	0.0076	−1.01	0.55
EG	0.18	0.55	−1.32	1.27	0.0100	−0.54	−0.59
MV	0.28	0.47	−1.13	1.10	0.0085	−0.56	−0.46
PV	0.29	0.52	−1.24	1.17	0.0094	−0.64	−0.55
LD	0.26	0.36	−0.94	0.97	0.0065	−0.48	−0.48
ID	0.25	0.46	−1.12	1.11	0.0084	−0.54	−0.52
PD	0.24	0.47	−1.05	1.14	0.0086	−0.53	−0.69
RB	0.17	0.39	−0.96	0.98	0.0071	−0.32	−0.62
CB	0.32	0.41	−1.03	1.05	0.0075	−0.62	−0.37
SIC	−0.64	0.46	−1.59	1.05	0.0084	0.50	−0.19
SID	0.49	0.26	0.00	1.79	0.0048	0.56	0.38
CLP	0.16	0.16	−0.41	0.53	0.0028	−0.52	−0.25

*WL–Wellness; DT–Discomfort Tolerance; PS–Problem-Solving; AE–Applying Experience; IG–Ingenuity; SU–Self-Understanding; SR–Self-Reliance; SC–Self-Control; CP–Coping; DC–Decisiveness; CR–Courage; SA–Social Awareness; CN–Connectedness; PT–Protectiveness; FM–Finding Meaning; EG–Engagement; MV–Motivation; PV–Perseverance; LD–Leadership; ID–Industriousness; PD–Productiveness; RB–Risk for Burnout; CB–Coachability; SIC–Self-Image Consistency; SID–Self-Image Desirability; CLP–Current Level of Performance. All scores were calculated after z-score conversion (SIA) and cube-root transformation (SIB).*

SDs are expected to lie in the 0.46–0.67 range based on core scale properties under the normal distribution curve after SIA and SIB are applied, with cube-root *z* minimum and maximum values lying near ±1.26 (2SD) to ±1.44 (3SD). Inherently, individuals differed most from one another in Discomfort Tolerance, and least in Finding Meaning. However, scale skewness and kurtosis fall well within acceptable ±2.0 ranges (Trochim and Donnelly, 2014). Small standard error of scale means suggests that similar results would be expected from larger samples obtained from the same population (Tabachnick and Fidell, 2001; Barde and Prajakt, 2012). Overall, the 18 MMP core scales demonstrated acceptable approximation of normal distribution patterns.

In Table 2, the core scales’ strength continues into the five ring scales, which underscores the MMP’s application strength. Their psychometric properties suggest that the MMP can extricate Leadership as a broad concept from performance, and that Industriousness and Productiveness are characteristically balanced in the normative population. While Risk for Burnout was low, the findings in Table 2 indicate that the MMP is able to detect when this risk becomes high. Similarly, the Coachability scale was able to demonstrate whether individuals are ready to commit to enhance their performance through self-development efforts.

Absolute differences in the responses to eight pairs of highly correlated items were low, indicating an acceptable level of consistency (SIC) in item responses by the majority of individuals in the normative population on which MMP validation was based. SID was expected to be somewhat elevated due to its nature, but scores lay within acceptable limits for most individuals. Many respondents had a realistic perception of their current level of performance (CLP), suggesting that it can be used as a reliable benchmark for identifying personal strengths and areas for further development to enhance performance.

### Demographic Impartiality of the MMP

We investigated the MMP’s classification effects (i.e., the extent to which its scales demonstrate stability across different demographic groups in the normative population). The data were grouped by: (a) gender in general and within broad occupational sectors; (b) age categories and generational cohorts; (c) educational levels; (d) managerial positions and responsibilities; and (e) citizenship. For the most part, we found no significant differences among comparable demographic subgroups, suggesting that the MMP offers widespread fairness and impartiality in assessing current performance between them. Gender differences had a small to medium effect, corresponding to approximately 1 T-score point difference (see Table 3). Males consistently scored higher than females in Coping with a medium effect size (differing by about 2 T-score points), and somewhat higher than females in Self-Control and Risk for Burnout, while females scored higher than males in Social-Awareness. One instance of suspected measurement invariance is that males consistently scored higher than females in Courage with a large effect size (differing by about 3 T-score points). Courage items could have been interpreted differentially, depending on whether work is office-based or requires bravery in the face of potential physical risk and danger. The latter was found to be more typical of male roles in specific occupations (Fiedeldey-Van Dijk and Bar-On, 2021). Therefore, while Courage appears to be gender inequivalent, context-sensitive interpretation allows for measurement precision in this performance factor.

**TABLE 3:** Gender comparison of MMP scale scores (*n_Males_* = 1,442; *n_Females_* = 1,185).

Factor /Scale	Mean Males	Mean Females	SD Males	SD Females	F (2-tailed)	Prob >F	Student t	Prob >| t|	Cohen’s *d*	Effect size
WL	−0.18	−0.22	0.56	0.55	1.03	0.58	1.65	0.10	0.06	Small
DT	0.23	0.21	0.63	0.68	1.17	0.00	1.00	0.32	0.04	Small
PS	0.30	0.24	0.44	0.46	1.10	0.10	3.22	0.00	0.13	Small
AE	0.49	0.46	0.42	0.44	1.10	0.09	2.19	0.03	0.09	Small
IG	0.28	0.23	0.42	0.44	1.05	0.37	2.83	0.00	0.11	Small
SU	0.21	0.18	0.51	0.53	1.08	0.17	1.63	0.10	0.06	Small
SR	−0.19	−0.04	0.60	0.60	1.02	0.78	−6.40	0.00	0.25	Medium
SC	0.13	−0.08	0.52	0.54	1.10	0.09	10.06	0.00	0.39	Medium
CP	0.23	0.07	0.44	0.49	1.19	0.00	8.61	0.00	0.34	Medium
DC	0.08	0.00	0.42	0.46	1.22	0.00	4.90	0.00	0.19	Medium
CR	0.36	−0.03	0.55	0.57	1.07	0.19	17.80	0.00	0.70	Large
SA	−0.13	0.14	0.54	0.58	1.13	0.02	−12.54	0.00	0.49	Medium
CN	0.16	0.32	0.54	0.50	1.16	0.01	−7.72	0.00	0.30	Medium
PT	0.03	−0.08	0.59	0.62	1.12	0.05	4.59	0.00	0.18	Medium
FM	0.44	0.53	0.42	0.41	1.07	0.22	−5.57	0.00	0.22	Medium
EG	0.20	0.19	0.52	0.57	1.18	0.00	0.42	0.67	0.02	Small
MV	0.26	0.30	0.47	0.47	1.01	0.85	−2.09	0.04	0.08	Small
PV	0.29	0.29	0.50	0.54	1.15	0.01	−0.01	0.99	0.00	Small
LD	0.32	0.21	0.35	0.35	1.02	0.70	8.01	0.00	0.31	Medium
ID	0.28	0.23	0.45	0.47	1.05	0.37	3.05	0.00	0.12	Small
PD	0.30	0.21	0.47	0.47	1.02	0.69	4.95	0.00	0.19	Medium
RB	0.25	0.12	0.38	0.39	1.07	0.23	8.70	0.00	0.34	Medium
CB	0.35	0.30	0.41	0.41	1.04	0.51	2.62	0.01	0.10	Small
SIC	0.48	0.51	0.26	0.26	1.03	0.54	−3.12	0.00	0.12	Small
SID	−0.71	−0.61	0.46	0.46	1.01	0.87	−5.61	0.00	0.22	Medium
CLP	0.18	0.16	0.16	0.15	1.12	0.05	4.16	0.00	0.16	Medium

*Underneath Table 2.*

We found MMP scale score differences between the youngest *versus* combined older age groups in the normative population, but not in wider generational cohorts. For example, those in their twenties performed significantly higher than those in their thirties up to fifties in Wellness and Motivation, and lower in Problem-Solving, Applying Experience, Ingenuity, Coping, Courage, and in all the ring scales (*p*<0.01). These differences appear to corroborate logical patterns among employees as they ascend the career ladder, suggesting that measurement invariance is not suspected. Therefore, the MMP appears to be widely applicable to all demographic groups.

### Reliability of the MMP

The internal consistency of MMP scales were examined based on the cube-root of *z*-score conversions of the item responses. Cronbach alphas need to be considered in relation to the number of items per scale, and the range within which individual items correlated with all scale items (see Table 4). Overall, the scales showed satisfactory reliability with internal consistency above 0.70 or close to it, which helps in achieving adequate scale validity as well (Hill and Lewicki, 2006). One exception was Self-Understanding. Subsequently, we added a new item to strengthen its reliability.

**TABLE 4:** Internal consistency of the MMP scales (*n* = 3,039).

Factor/Scale	Number of items	Cronbach’s coefficient alpha	Lowest item-scale correlation	Highest item-scale correlation
Wellness	7	0.75	0.23	0.67
Discomfort Tolerance	4	0.71	0.41	0.56
Problem-Solving	6	0.64	0.27	0.46
Applying Experience	7	0.72	0.33	0.58
Ingenuity	9	0.76	0.35	0.52
Self-Understanding	4(1)	0.51	0.20	0.38
Self-Reliance	5	0.72	0.35	0.59
Self-Control	5	0.68	0.36	0.54
Coping	7	0.71	0.39	0.50
Decisiveness	7	0.60	0.26	0.44
Courage	5	0.72	0.38	0.57
Social-Awareness	5	0.71	0.38	0.61
Connectedness	5	0.67	0.33	0.47
Protectiveness	4	0.65	0.32	0.55
Finding Meaning	6	0.65	0.29	0.44
Engagement	6	0.74	0.34	0.62
Motivation	6	0.62	0.25	0.47
Perseverance	5	0.70	0.39	0.60
Leadership	18(1)	0.79	0.27	0.51
Industriousness	4(4)	-	-	-
Productiveness	4(7)	-	-	-
Risk for Burnout	10	0.68	0.24	0.45
Coachability	7(1)	0.66	0.32	0.40
Self-Image Desirability	6	0.66	0.31	0.49

*Number of items are based on highest factor loadings; new items in parentheses were excluded from calculations; Cronbach alphas for Industriousness and Productiveness are omitted due to partial data. Underneath Table 2.*

The impact of comparatively lower scale SD and maximum scores on reported Cronbach alphas deserve scrutiny (see Table 2). Scales with a more pronounced negative skew and addressed with SIB could result in a Cronbach alpha below 0.70 resulting from a restriction in range of variation. This would depress their value (Fife et al., 2012) and support a conservative presentation of their internal consistency compared to conventional presentations where acquiescence bias is not methodologically addressed. Ring scales demonstrated satisfactory internal consistency as well, even when items were originally developed for the core scales. Leadership, in particular, achieved high internal consistency, partially as a result of containing a higher number of items than other scales to broadly represent leadership performance.

Inter-correlation coefficients between MMP scales were expected to range between −1 to +1 for *z*-scored items (based on applying SIA). Correlations between scales lay moderately around 0 in the matrix, with 18 out of 153 possible scale pairs correlating above or below 0.30. Scale pairs of Problem-Solving and Ingenuity, and Coping and Courage had the strongest inter-scale correlations with 0.47 each. As was expected, correlations between ring scales were stronger, varying from 0.47-0.73. Three of the 10 possible pairs were >0.70, with Industriousness correlating the lowest with other ring scales. Overall, the reliability pattern across MMP scales demonstrated satisfactory strength, which underscores that MMP factors provide meaningful perspectives on performance for application purposes.

### Discriminant and Predictive Strength of the MMP

We rescored the MMP responses of 114 certified emergency managers and 215 emergency managers who were not certified from data provided by Murphy (2018). It was found that the certified group scored significantly higher in Ingenuity, Problem-Solving, Applying Experience, and Protectiveness than the non-certified group (*p*<0.05; see Table 5). The certified group also scored comparatively higher in Leadership, Productiveness, and Coachability. The certified group further outperformed the non-certified group in Connectedness and Engagement (*p*<0.10), indicating that the MMP widely discriminates between performance factors. Interpretationally, the statistical significance of medium effect size corresponds with a difference between the two groups of approximately 2-3 T-score points. The estimated magnitude of this difference matters when performance compliance in this occupation is generally high, as indicated by the relatively small MMP scale SDs compared to the normative population in Table 2.

**TABLE 5:** Differences in MMP scale scores between certified (CEM) and non-certified (Non) emergency managers (*n*_*CEM*_=114; *n*_*Non*_=215).

Factor /Scale	Mean CEM	Mean Non	SD CEM	SD Non	F (2-tailed)	Prob >F	Student t	Prob >| t|	Cohen’s *d*	Effect size
WL	−0.41	−0.23	0.49	0.53	1.13	0.46	2.94	0.00	0.34	Medium
DT	0.36	0.35	0.53	0.59	1.26	0.17	−0.19	0.85	0.02	Small
PS	0.56	0.43	0.34	0.35	1.09	0.61	−3.22	0.00	0.38	Medium
AE	0.65	0.57	0.32	0.31	1.05	0.77	−2.18	0.03	0.25	Medium
IG	0.52	0.40	0.28	0.33	1.38	0.06	−3.19	0.00	0.38	Medium
SU	0.21	0.19	0.46	0.49	1.11	0.54	−0.37	0.71	0.04	Small
SR	−0.29	−0.27	0.52	0.55	1.11	0.53	0.33	0.75	0.04	Small
SC	0.23	0.14	0.50	0.44	1.29	0.12	−1.62	0.11	0.18	Medium
CP	0.40	0.34	0.34	0.38	1.25	0.19	−1.59	0.11	0.19	Medium
DC	0.19	0.13	0.33	0.39	1.38	0.06	−1.42	0.16	0.17	Medium
CR	0.44	0.47	0.45	0.48	1.10	0.56	0.48	0.63	0.06	Small
SA	−0.13	−0.16	0.52	0.52	1.01	0.98	−0.53	0.60	0.06	Small
CN	0.14	0.24	0.51	0.48	1.13	0.43	1.73	0.08	0.20	Medium
PT	0.26	0.16	0.47	0.54	1.32	0.10	−1.70	0.09	0.20	Medium
FM	0.55	0.55	0.31	0.33	1.09	0.61	−0.04	0.97	0.00	Small
EG	0.39	0.31	0.38	0.46	1.51	0.01	−1.65	0.10	0.20	Medium
MV	0.21	0.26	0.45	0.47	1.10	0.58	0.99	0.32	0.12	Small
PV	0.35	0.37	0.42	0.46	1.21	0.27	0.46	0.65	0.05	Small
LD	0.54	0.45	0.23	0.26	1.35	0.07	−3.04	0.00	0.36	Medium
ID	0.44	0.40	0.35	0.38	1.17	0.35	−1.06	0.29	0.12	Small
PD	0.51	0.42	0.30	0.38	1.56	0.01	−2.30	0.02	0.26	Medium
RB	0.40	0.36	0.28	0.28	1.04	0.81	−1.08	0.28	0.13	Small
CB	0.54	0.47	0.32	0.31	1.05	0.76	−2.10	0.04	0.24	Medium
SIC	0.47	0.48	0.24	0.24	1.06	0.69	0.27	0.78	0.03	Small
SID	−0.87	−0.82	0.35	0.38	1.18	0.32	1.04	0.30	0.12	Small
CLP	0.26	0.24	0.09	0.10	1.41	0.04	−2.07	0.04	0.25	Medium

*Underneath Table 2.*

We rescored the MMP responses of 430 North American law enforcement officers that Conroy (2017) collected together with scores from the Multifactor Leadership Questionnaire (Bass and Avolio, 1990, 1995). This was done to investigate how well the MMP could predict transformational, transactional, and passive-avoidant leadership styles. The results in Table 6 indicate that the MMP core scales achieved comparable strength in model fit in predicting leadership regardless of style, yet with a different combination of performance factors. Specifically, most core factors contribute robustly to transformational leadership (*R*^2^=68.95).

**TABLE 6:** Predicting different leadership styles with the MMP (*n* = 430).

Factor/ Scale	Transformational leadership *R*^2^ = 0.6895				Transactional leadership *R*^2^ = 0.6634				Passive-Avoidant leadership *R*^2^ = 0.5462			
	β	SE	F	p	β	SE	F	p	β	SE	F	p
Intercept	4.17	0.03	24,790.40	0.00	4.47	0.03	17,432.30	0.00	4.55	0.03	22,576.90	0.00
WL	0.04	0.02	5.08	0.02	0.16	0.02	51.00	0.00	−0.07	0.02	9.83	0.00
DT	−0.04	0.02	5.74	0.02	−0.05	0.02	5.71	0.02	−0.06	0.02	7.23	0.01
PS									0.07	0.03	5.31	0.02
AE	−0.07	0.02	14.92	0.00	−0.03	0.02	1.47	0.23	0.02	0.03	0.78	0.38
IG	−0.07	0.02	7.53	0.01	−0.06	0.03	3.26	0.07	−0.04	0.03	1.52	0.22
SU	−0.36	0.01	621.79	0.00	−0.37	0.02	407.23	0.00	−0.34	0.02	389.77	0.00
SR	−0.01	0.02	0.53	0.47	−0.07	0.02	8.49	0.00	−0.03	0.02	1.46	0.23
SC	−0.07	0.02	14.40	0.00	−0.05	0.02	3.91	0.05	0.04	0.02	2.74	0.10
CP	0.07	0.03	6.70	0.01	0.03	0.04	0.71	0.40				
DC	0.07	0.03	6.08	0.01	−0.04	0.03	1.58	0.21				
CR	−0.15	0.03	25.92	0.00	−0.14	0.04	13.87	0.00	−0.15	0.04	16.68	0.00
SA	0.08	0.02	16.05	0.00	0.03	0.03	1.35	0.25	0.03	0.02	1.53	0.22
CN	−0.02	0.02	1.39	0.24	−0.06	0.02	6.54	0.01	−0.05	0.02	5.20	0.02
PT	0.07	0.02	12.14	0.00	0.16	0.03	34.23	0.00	0.06	0.03	4.51	0.03
FM	−0.08	0.02	9.88	0.00	−0.10	0.03	11.07	0.00				
EG	0.03	0.02	2.34	0.13	−0.05	0.03	2.76	0.10	−0.02	0.03	0.81	0.37
MV	0.12	0.02	31.01	0.00	0.22	0.03	61.14	0.00	0.03	0.03	1.44	0.23
PV	−0.05	0.02	5.66	0.02	−0.04	0.03	1.79	0.18				

*Underneath Table 2. β–parameter estimates; SE–Standard error of estimates; F–F value, ratio of the regression mean square to the error mean square; p–significance probability of F value; R^2^–predictive power, square of the multiple correlation coefficient.*

Notably, all the MMP scales that assess performance explain the three leadership styles in law enforcement, although somewhat uniquely in scale contributions. Leadership in the transformational style was characterized by high Motivation and Social-Awareness, with low Applying Experience and Self-Control. By comparison, in the transactional style, high Motivation and Wellness emerged with low Self-Reliance. Law-enforcement officers leading with a passive-avoidant style demonstrated high Problem-Solving with low Wellness. High Protectiveness with low Self-Understanding and Courage among law-enforcement officers contributed significantly to all leadership styles.

We also explored the concurrent power of the core scales to predict each ring scale separately using the normative population (see Tables 7, 8). Ring scales are independent from most core scales with a few exceptions of partial dependency. They sparingly share items in a way that is not specific to any particular scale. By design, ring scales purposely lie outside of Bar-On’s core factor model of human performance. No significant portion of any core scale is nested within a ring scale, and the degree of core-ring scale dependence is negligible. The predictive strength of the core scales ranged from 55 to 92% when modeled against the ring factors. The core scale combinations suggest that the MMP is well positioned for top performer benchmarking for the ring factors.

**TABLE 7:** Predicting Industriousness and Productiveness with the MMP (*n* = 3,039).

Factor/ Scale	Industriousness *R*^2^ = 0.5541				Productiveness *R*^2^ = 0.6540			
	β	SE	F	p	β	SE	F	p
Intercept	−0.02	0.01	1.82	0.18	−0.02	0.01	3.38	0.07
WL	−0.01	0.01	1.17	0.28	−0.01	0.01	1.58	0.21
DT	0.04	0.01	18.69	0.00				
PS	0.06	0.01	13.78	0.00	0.07	0.01	24.38	0.00
AE	0.02	0.01	1.79	0.18	0.03	0.01	7.09	0.01
IG	0.35	0.02	438.11	0.00	0.67	0.01	2,023.53	0.00
SU	−0.01	0.01	1.64	0.20	−0.01	0.01	1.46	0.23
SR	0.04	0.01	18.06	0.00				
SC	−0.02	0.01	2.82	0.09	0.04	0.01	13.09	0.00
CP	0.30	0.02	410.83	0.00	0.22	0.01	232.85	0.00
DC	0.03	0.01	3.12	0.08	0.05	0.01	13.52	0.00
CR					−0.02	0.01	3.02	0.08
SA					0.02	0.01	2.55	0.11
CN	−0.02	0.01	2.66	0.10	−0.01	0.01	0.47	0.49
PT	−0.02	0.01	5.96	0.01	−0.01	0.01	2.00	0.16
FM	0.03	0.01	4.95	0.03	0.03	0.01	6.35	0.01
EG	0.02	0.01	2.76	0.10	0.04	0.01	13.83	0.00
MV	−0.03	0.01	5.13	0.02	−0.04	0.01	10.80	0.00
PV	0.36	0.01	837.41	0.00	0.08	0.01	47.37	0.00

*Data for Industriousness and Productiveness as criterion variables are incomplete. Underneath Table 2, and underneath Table 6.*

**TABLE 8:** Predicting Leadership, Risk for Burnout and Coachability with the MMP (*n* = 3,039).

Factor/ Scale	Leadership *R*^2^ = 0.9148				Risk for Burnout *R*^2^ = 0.8610				Coachability *R*^2^ = 0.7991			
	β	SE	F	p	β	SE	F	p	β	SE	F	p
Intercept	0.03	0.00	62.57	0.00	0.01	0.01	7.15	0.01	−0.03	0.01	22.31	0.00
WL					0.01	0.01	1.26	0.26				
DT	0.01	0.00	9.87	0.00	0.00	0.00	0.92	0.34	0.03	0.01	26.88	0.00
PS	0.15	0.01	929.42	0.00	−0.02	0.01	5.20	0.02	0.16	0.01	342.45	0.00
AE	0.06	0.00	150.04	0.00	0.02	0.01	7.76	0.01	0.33	0.01	1,542.47	0.00
IG	0.40	0.01	5,104.84	0.00	0.36	0.01	2,325.99	0.00	0.58	0.01	3,463.04	0.00
SU	−0.02	0.00	25.47	0.00	−0.02	0.01	10.80	0.00	−0.02	0.01	9.61	0.00
SR	0.00	0.00	1.74	0.19	0.01	0.00	1.96	0.16				
SC	−0.01	0.00	3.32	0.07	0.35	0.01	3,813.02	0.00				
CP	0.26	0.01	2,393.04	0.00	0.31	0.01	1,764.28	0.00	0.01	0.01	0.60	0.44
DC	0.12	0.00	635.83	0.00					0.01	0.01	2.23	0.14
CR	0.10	0.00	537.61	0.00	−0.01	0.01	4.91	0.03	−0.01	0.01	4.39	0.04
SA	0.00	0.00	0.75	0.39								
CN	0.00	0.00	1.14	0.29	0.01	0.01	2.21	0.14	0.01	0.01	1.98	0.16
PT	−0.02	0.00	23.41	0.00	0.02	0.00	23.72	0.00				
FM	0.01	0.00	2.27	0.13	0.03	0.01	22.67	0.00	0.02	0.01	7.33	0.01
EG	0.00	0.00	0.79	0.37					−0.02	0.01	10.09	0.00
MV	−0.02	0.00	11.54	0.00					−0.04	0.01	18.76	0.00
PV	0.01	0.00	8.48	0.00	0.01	0.01	4.07	0.04	0.02	0.01	9.54	0.00

*Underneath Tables 2, 6.*

The Industriousness and Productiveness ring scales describe the interdependent relationship of working both hard and smart, to which high Problem-Solving and Coping contributed significantly (see Table 7). While Perseverance and, especially, Ingenuity contributed to both work approaches, the former was particularly significant for predicting Industriousness, and the latter for predicting Productiveness. Other contributors to Industriousness were Discomfort Tolerance and Self-Reliance, while Decisiveness further added to Productiveness.

In Table 8, wide scale contributions are evident for the three remaining ring scales. The MMP core scale with the largest number of items, Ingenuity, emerged as a key predictor of performance in ring factors. Furthermore, strong Leadership performance was explained by high core scale scores in general, and notably by Coping, Problem-Solving, Decisiveness, Courage, and Applying Experience. Risk for Burnout, which was assessed before COVID-19, was found to be driven by elevated levels of Ingenuity, Self-Control, Coping, and Finding Meaning to a lesser extent. Re-assessment with enduring epidemiological conditions of stress might bring additional core scales to light, such as Applying Experience, Problem-Solving, and Courage. The Coachability scale was most strongly impacted by high Ingenuity, Applying Experience, Problem-Solving, and Discomfort Tolerance, with low Motivation.

The demonstrated validity and practical significance, based on three different studies presented here, suggest that performance assessment with the MMP will yield accurate results that matter in the workplace.

## Discussion

Cronbach (1988) and Messick (1988) argued that the value of assessments needs to be infused with statistical demonstrations of accuracy, which are emphasized in the Classical Validity Framework as described by Cronbach and Meehl (1955). More recently, Gregory (1992) and Messick (1995) proposed a Unified Validity Framework. This validation process was jointly endorsed by three professional associations (American Educational Research Association [AERA], American Psychological Association [APA], and National Council on Measurement in Education [NCME], 1999). In the Unified Validity Framework, validation is an integrated and evaluative process based on two requirements (Cronbach, 1982, 1988; Messick, 1989; Strauss and Smith, 2009). First, it needs to demonstrate classical validation evidence of a psychometric assessment underpinned by theory. Second, it needs to argue for and support the adequacy, as well as appropriateness of interpretation and applications based on the findings of that assessment, including their possible limitations and consequences.

The process is essentially an integrated and evaluative judgment anchored in six complementary and pragmatic aspects, which are offered as general validity criteria with associated *standards* to be considered in the context of their potential repercussions (American Educational Research Association [AERA], American Psychological Association [APA], and National Council on Measurement in Education [NCME], 1999). Each aspect elicits a pertinent question that can be demonstrated in several ways by the Bar-On model and measure of performance, for example. The systematic scrutiny of MMP characteristics and application features, examined below, shows that they fit well within the Unified Validity Framework.

### Content Relevance and Representativeness

• *Does the MMP content measure performance, and can it do so consistently?*

The MMP assessment consists of 120 unique items that measure performance based on current behavior, using a 9-point rating scale as described in Subsection “Development of an Advanced Response Scale for the MMP.” These items have strong psychometric properties after weaker items were progressively eliminated over an eight-year period. Their refined wording was evaluated as being *neutral* with independent testing for text readability (Child, 2020). The MMP factor descriptions presented in Subsection “Core Factors Assessed with the MMP” closely reflect their corresponding scale and item content to enhance content precision. The accuracy of factor interpretations, as assessed by the scales, is further enhanced by descriptors that cover a continuum of score ranges positioned under the normal distribution curve.

End-users, representing different demographic subgroups, frequently report that their assessment results describe them accurately. This feedback anecdotally supports overall performance consistency in diverse conditions as described in Subsection “Demographic Impartiality of the MMP.” The normative population is becoming increasingly etic in character and representativeness, which will lead to more studies to empirically demonstrate measurement invariance.

Individuals’ responses to the assessment are subject to a novel scoring algorithm as described in Subsection “Scoring Algorithm of the MMP” that includes T-score standardization. This process was created to establish a baseline to more realistically measure current performance for multiple applications in a reliable manner.

### Response Processes and Regularity

• *Is the theoretical foundation sound as captured by the MMP scales?*

The MMP factor and scale development was substantive (see Subsection “Scale and Item Refinement of the MMP”), which established and supported a comprehensive model of human performance. This was intended by design and as reflected by the full name of the assessment, the Bar-On Multifactor Measure of Performance. It could be argued that Bar-On’s approach to initially selecting potential key contributors to performance from the literature is subjective, and that other researchers might have reviewed the literature differently, selected other factors and/or described them in another manner. However, the empirical validation described in Section “Results” supports his model.

**FIGURE 1:**
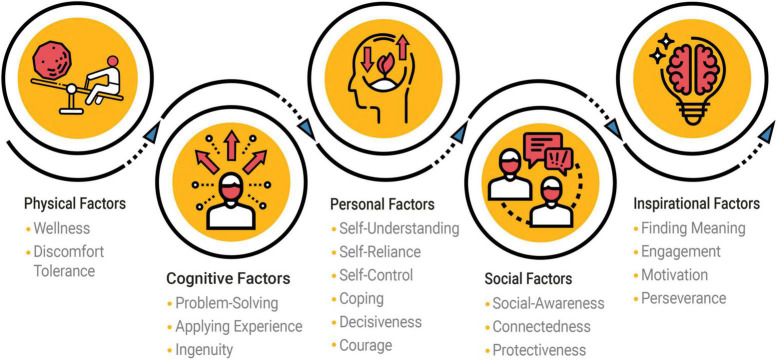
Conceptual presentation of the MMP model, supported by principal components. © 2021 Into Performance ULC. All Rights Reserved.

The factors were refined through the development of four versions of the MMP and are presently assessed with 18 psychometric scales. These are grouped into five categories as shown in Figure 1. The principal components showed a good fit with 46.32% of the variance in performance explained. PCA results, which were based on an English-speaking normative population of size 3,039 that was 71.77% North American and demographically balanced, showed that performance consists of multiple factors.

The MMP scales demonstrated satisfactory reliability based on Cronbach’s alpha scores of approximately 0.70, which were achieved after *z*-score conversion (SIA) and cube-root transformation (SIB) of the item responses. We expect that internal consistency will increase somewhat in strength following the addition of 13 new items, and as a result of the wording refinement that some items underwent to strengthen them. The MMP’s item and scale properties as described in Subsection “Psychometric Properties of the MMP Scales” meet American Educational Research Association [AERA], American Psychological Association [APA], and National Council on Measurement in Education [NCME] (1999) development standards. The flat structure of the performance model enables the integration with related competency frameworks that some organizations apply.

### Scoring and Structural Alignment

• *Do the MMP scales correlate with each other, and are they realistic upon reflection?*

The MMP’s psychometrically demonstrated reliability and validity present a realistic picture of scientific strength. Common issues with response bias when using self-rated assessments, which are typically left to the practitioner’s interpretation of the results, are addressed in the MMP through cube-root *z*-score adjustment in its scoring algorithm to enable fair comparison between individual and group results, as described in Subsection “Scoring Algorithm of the MMP.” The built-in indices of response consistency (SIC) and desirability (SID), as well as other indicators, help strengthen the integrity of results, and alert practitioners to take appropriate action when credibility is in question as described in Subsection “Additional Metrics of the MMP.”

The MMP’s scales are low to moderately correlated, indicating that they are part of the overarching construct of performance as described in Subsection “Reliability of the MMP.” At the same time, the scales are sufficiently distinct from one another to facilitate meaningful interpretations in making better selection, placement, and development decisions.

MMP results also include a measure of the individual’s perceived current level of performance (CLP) as described in Subsection “Additional Metrics of the MMP.” CLP correlated significantly with average scale performance for approximately four out of five individuals in the normative population. CLP correlated positively with all core scales with an average of 0.30. The correlation was lowest between CLP and Self-Reliance, and highest was between CLP and Ingenuity. The average correlation between CLP and the ring scales was 0.61. This indicates that, apart from associations with underlying core factors, ring factors add meaning to debriefing, hiring and coaching discussions of performance.

### Generalizability and Fairness

• *Does the MMP generalize across different groups, settings and circumstances?*

The assessment’s large, composite and diverse normative population contributed to determining a performance factor structure that is suitable for use universally. It enabled the establishment of a general norm whereby results can be presented in a standardized fashion by scoring default (SIE) as described in Subsection “Scoring Algorithm of the MMP.” Demographic subgroups scored well within 2.5 T scores from the average T score of 50 on the MMP scales, with a standard deviation of 10. In other words, normative subgroups perform comparably for the most part.

The interpretation of MMP results is facilitated by a number of different norms to enhance precision in context-specific interpretation when needed, as characterized by specific demographics. For example, a specialized norm might be more appropriate than a general norm when there is cause for gender or occupational equity, or when a young adult joins a team comprising older colleagues, as described in Subsection “Demographic Impartiality of the MMP.” This assessment is able to differentiate between groups based on distinct factors that contribute to performance. The description of workplace performance with ring scales indicates that scores tend to increase with age, higher educational levels, and higher job positions.

Two independent studies described in Subsection “Discriminant and Predictive Strength of the MMP” demonstrated the MMP’s discriminant strength for leadership styles and job-relevant training. The MMP, and the construct it assesses, will benefit from continued validation based on researching large and diverse samples across cultures and in different settings. For example, future scale analyses will help confirm its factorial structure, validity, and internal consistency. Test-retest reliability, additional predictive and incremental validity studies, as well as other statistical examinations are currently being planned.

### Relation to Other Variables

• *Does the MMP have convergent, discriminant and predictive strength?*

Significant events or impactful situations can affect current performance negatively. The MMP assessment includes seven statements that describe the degree to which such possibilities might have occurred, using the same 9-point response scale that is applied to rating scale items. This information will enable refined validation efforts in future studies. These situational responses could also help us better understand measurement invariance under conditions of particular personal and social challenges.

A growing number of researchers conduct studies in various industry sectors on topics of special interest, such as leadership, resilience, remote-hybrid work conditions, burnout, and others. The core scales have moderate success in predicting well-being indicators such as occupational stress, job satisfaction and happiness, which are notoriously evasive.

While the MMP scales were found to predict different leadership styles and to profile them uniquely as described in Subsection “Discriminant and Predictive Strength of the MMP,” additional studies that include an external criterion to demonstrate relevance in different applications will be helpful. Studies that include external psychometric assessments will need to be conducted to learn more about its convergent and discriminant validity.

### Intended and Unintended Consequences

• *Does the MMP have merit despite the potential risk for invalid scores or inappropriate interpretation?*

The Bar-On Multifactor Measure of Performance (MMP) is published by Into Performance ULC in Canada, and administered online *via* the mmp2perform.com website. The assessment can be reliably completed from any electronic device. This assessment is classified as a level-B psychometric test, and administered *via* secure dashboard access. The successful completion of an accreditation workshop qualifies individuals to interpret and debrief the results.

This assessment is applicable for assessing individuals 18 to 80 years of age. The readability of its scale descriptions was evaluated at the 14.7 Flesch-Kincaid grade level (Child, 2020). By comparison, readability of the assessment items was rated at grade level 6.7. This means that those whose reading skills are between the 8^th^ and 10^th^ grade are expected to easily understand the wording of the items. Item content is conversational rather than formal, and phrased in the positive (Child, 2020). Average completion time is approximately 20 minutes.

The MMP generates report sets that address a variety of business functions and operational needs at individual, group and organizational levels. Online reporting enables multiple layers of support, guidelines and directives for accurate interpretation of profiled results. While the MMP’s general norm is applicable for use with most reports, several demographic-specific norms and different benchmarks are available as well. Results are presented with online interactivity in graphical, numerical and textual formats. Development reports provide practical suggestions for strengthening performance, together with an electronic Personal Workbook and Activity Journal to monitor progress.

## Conclusion

In this article, we described the ongoing validation of a multifactor conceptual model and psychometric instrument – the MMP – that is designed to study, assess and enhance human performance. The key findings presented in this article suggest that we have adequately addressed the need for a multifactor model and assessment of performance, which was scientifically developed and validated. A key advantage of this measure is that it can assess performance comprehensively in one sitting, effectively reducing the cost of conducting a number of different tests needed to evaluate one’s current level of performance.

It was shown that this conceptual and psychometric model was methodically developed based on a systematic search of the literature, input from expert consultants, as well as a rigorous application of descriptive, inferential and multivariate statistics, designed to examine its validity, reliability and application. We demonstrated how the MMP is capable of concomitantly assessing 18 Core Factors grouped into *physical*, *cognitive*, *personal*, *social*, and *inspirational* factor categories, as well as 5 additional Ring Factors that assess *leadership*, *industriousness*, *productiveness*, *risk for burnout*, and *coachability*, in the workplace and elsewhere.

In addition to its psychometric strength, it was also explained that the MMP is solution-oriented in offering development suggestions for strengthening underdeveloped contributors to performance when scale scores indicate a deviation from a set benchmark. Thus, this model was designed to help understand why some people perform better than others as well as what needs to be focused on to improve individual and organizational performance.

The MMP is designed and positioned for a full-range of Human Resource functions such as the following: Career counseling; recruitment and selection; on- and off-boarding; self-development; coaching and mentoring; team building; and succession planning. It is also suitable for single and repeated administration as circumstances change, such as organizational restructuring and, especially, for tracking progress following training and coaching. Additionally, this model is potentially applicable in parenting, healthcare, education, as well as in researching multiple aspects of human performance.

Against the backdrop of continued psychometric strengthening, this approach to conceptualizing human performance and the systematic method of assessing it has been adequately demonstrated, iteratively tested, and empirically examined to be applied with confidence by accredited professionals.

## Data Availability Statement

The data analyzed in this study is subject to a copyright license with restricted access. The authors own the datasets. Researchers interested in viewing them, beyond the Supplementary Material provided, will need to receive the authors’ permission. Requests to view these datasets should be directed to the corresponding author.

## Ethics Statement

Ethical review, approval and written informed consent for participation were not required for this study on human participants, in accordance with the local legislation and institutional requirements.

## Author Contributions

Both authors listed have made a substantial, direct, and intellectual contribution to the work, and approved it for publication.

## Conflict of Interest

The authors declare that the research was conducted in the absence of any commercial or financial relationships that could be construed as a potential conflict of interest.

## Publisher’s Note

All claims expressed in this article are solely those of the authors and do not necessarily represent those of their affiliated organizations, or those of the publisher, the editors and the reviewers. Any product that may be evaluated in this article, or claim that may be made by its manufacturer, is not guaranteed or endorsed by the publisher.
