# Supplementary material for: The Bar-On Model and Multifactor Measure of Human Performance: Validation and Application
Source: Front Psychol. 2022 Jul 4;13:872360. doi: 10.3389/fpsyg.2022.872360 (PMC9291401; doi:10.3389/fpsyg.2022.872360)
Supplement: Supplementary file 1 [file Data_Sheet_1.pdf]

Supplementary Material

Root Mean Square Off-diagonal Residuals: Over-all = 0.03029342

ITEM001	ITEM004	ITEM005	ITEM007	ITEM008	ITEM009	ITEM011	ITEM012	ITEM015	ITEM016	ITEM018
0.027173	0.032359	0.034461	0.032073	0.029159	0.030128	0.033486	0.032364	0.029524	0.031729	0.032118
ITEM019	ITEM021	ITEM022	ITEM023	ITEM026	ITEM027	ITEM029	ITEM030	ITEM033	ITEM034	ITEM037
0.024162	0.034904	0.032979	0.034486	0.029923	0.028654	0.033142	0.035316	0.030200	0.027889	0.023735
ITEM038	ITEM039	ITEM040	ITEM041	ITEM042	ITEM043	ITEM044	ITEM045	ITEM046	ITEM049	ITEM050
0.030875	0.030748	0.029672	0.033372	0.030626	0.030745	0.032141	0.032852	0.029580	0.025385	0.033348
ITEM051	ITEM052	ITEM053	ITEM054	ITEM055	ITEM056	ITEM057	ITEM058	ITEM059	ITEM060	ITEM061
0.032286	0.030419	0.028460	0.029694	0.032151	0.029674	0.026296	0.030014	0.034031	0.031647	0.026153
ITEM062	ITEM063	ITEM064	ITEM065	ITEM068	ITEM069	ITEM071	ITEM072	ITEM073	ITEM074	ITEM075
0.032681	0.028255	0.028825	0.029639	0.029143	0.033915	0.033330	0.028807	0.030358	0.028164	0.029359
ITEM076	ITEM077	ITEM079	ITEM080	ITEM081	ITEM082	ITEM083	ITEM084	ITEM085	ITEM087	ITEM088
0.033751	0.023899	0.031894	0.028775	0.029470	0.029984	0.034222	0.030856	0.029980	0.027315	0.028867
ITEM089	ITEM090	ITEM091	ITEM092	ITEM093	ITEM094	ITEM095	ITEM097	ITEM098	ITEM100	ITEM102
0.028912	0.027176	0.031447	0.031548	0.028312	0.027237	0.029497	0.029891	0.028044	0.030633	0.030852
ITEM103	ITEM105	ITEM106	ITEM108	ITEM109	ITEM113	ITEM114	ITEM115	ITEM116	ITEM117	ITEM118
0.026763	0.032661	0.027610	0.028570	0.028250	0.032680	0.032865	0.028102	0.031970	0.028504	0.030758
ITEM119	ITEM120	ITEM121	ITEM123	ITEM124	ITEM125	ITEM128	ITEM131	ITEM135	ITEM137	ITEM138
0.027425	0.029369	0.031661	0.032340	0.028916	0.030715	0.029633	0.027876	0.034102	0.028498	0.030344
ITEM139	ITEM140									
0.027432	0.027220									

Root Mean Square Off-diagonal Partials: Over-all = 0.05786702

ITEM001	ITEM004	ITEM005	ITEM007	ITEM008	ITEM009	ITEM011	ITEM012	ITEM015	ITEM016	ITEM018
0.058575	0.055461	0.065291	0.059575	0.056565	0.065821	0.062155	0.060030	0.063375	0.059471	0.055598
ITEM019	ITEM021	ITEM022	ITEM023	ITEM026	ITEM027	ITEM029	ITEM030	ITEM033	ITEM034	ITEM037
0.059656	0.066513	0.056652	0.064989	0.059240	0.064798	0.063498	0.063753	0.057512	0.062447	0.057085
ITEM038	ITEM039	ITEM040	ITEM041	ITEM042	ITEM043	ITEM044	ITEM045	ITEM046	ITEM049	ITEM050
0.059678	0.060159	0.051306	0.057470	0.053645	0.053879	0.058634	0.060253	0.050145	0.056019	0.068447
ITEM051	ITEM052	ITEM053	ITEM054	ITEM055	ITEM056	ITEM057	ITEM058	ITEM059	ITEM060	ITEM061
0.060594	0.060581	0.052773	0.054908	0.055168	0.058496	0.059011	0.059228	0.058929	0.058308	0.057956
ITEM062	ITEM063	ITEM064	ITEM065	ITEM068	ITEM069	ITEM071	ITEM072	ITEM073	ITEM074	ITEM075
0.065884	0.051042	0.054018	0.051317	0.053072	0.064228	0.059771	0.060398	0.059085	0.057280	0.054740
ITEM076	ITEM077	ITEM079	ITEM080	ITEM081	ITEM082	ITEM083	ITEM084	ITEM085	ITEM087	ITEM088
0.061603	0.055086	0.059778	0.059735	0.054304	0.054354	0.061108	0.068704	0.050993	0.054961	0.049894
ITEM089	ITEM090	ITEM091	ITEM092	ITEM093	ITEM094	ITEM095	ITEM097	ITEM098	ITEM100	ITEM102
0.049721	0.050850	0.058488	0.055840	0.057603	0.054366	0.056558	0.063114	0.048930	0.059017	0.054681
ITEM103	ITEM105	ITEM106	ITEM108	ITEM109	ITEM113	ITEM114	ITEM115	ITEM116	ITEM117	ITEM118
0.063221	0.057479	0.052363	0.049078	0.049433	0.054671	0.059630	0.060905	0.058337	0.052249	0.058321
ITEM119	ITEM120	ITEM121	ITEM123	ITEM124	ITEM125	ITEM128	ITEM131	ITEM135	ITEM137	ITEM138
0.057007	0.053816	0.061013	0.057349	0.052913	0.065601	0.056305	0.051233	0.057356	0.061776	0.054342
ITEM139	ITEM140									
0.058753	0.048896									

Principal Components Analysis - Rotation Method: Varimax

Rotated Factor Pattern

	FACTOR1	FACTOR2	FACTOR3	FACTOR4	FACTOR5
ITEM090	0.61754	0.02139	0.02394	-0.01791	0.04045
ITEM044	0.58924	0.13910	-0.02429	-0.06096	-0.00330
ITEM089	0.56121	0.07281	-0.00343	-0.04277	0.00945
ITEM045	0.55486	0.10989	-0.04211	-0.05531	0.00964
ITEM065	0.54468	-0.06710	0.08306	-0.05033	-0.04896
ITEM043	0.52685	-0.04087	-0.04923	-0.05695	0.00499
ITEM042	0.49195	-0.05173	0.11604	-0.02109	-0.03077
ITEM088	0.47670	0.05768	0.22937	-0.03338	-0.02762
ITEM108	0.42927	0.12026	0.06741	-0.03958	0.10862
ITEM046	0.39747	0.06479	0.07468	-0.01036	-0.05200
ITEM109	0.37061	0.06452	0.06111	-0.00876	0.03687
ITEM119	-0.02375	0.72156	-0.02704	0.01717	-0.03213
ITEM056	-0.04561	0.69366	-0.04204	0.03052	-0.09702
ITEM033	0.06411	0.58431	-0.05638	0.10690	0.12070
ITEM140	0.11951	0.55918	-0.03407	-0.00413	0.08869
ITEM098	0.07983	0.54955	0.08054	-0.00916	0.16235
ITEM120	0.09617	0.54284	0.07972	-0.04747	-0.00711
ITEM053	-0.00627	0.51165	0.03858	0.01467	0.17583
ITEM011	-0.01283	0.49254	-0.04187	0.07850	0.11806
ITEM055	0.05633	0.44880	0.10487	0.02688	0.09899
ITEM074	0.07293	0.01491	0.68594	0.04498	-0.03443
ITEM052	0.11415	-0.02788	0.64313	-0.04309	-0.01945
ITEM051	0.02450	-0.00587	0.63412	0.00215	-0.04193
ITEM094	-0.04176	0.04791	0.62354	0.01425	-0.05076
ITEM075	0.28487	-0.00510	0.53909	-0.08084	-0.03151
ITEM029	0.13179	-0.04312	0.47368	0.06438	-0.05504
ITEM116	-0.04648	-0.03861	0.44903	0.01983	0.05077
ITEM138	-0.09773	0.15436	0.42140	0.20789	-0.01140
ITEM019	-0.06153	0.03170	0.02952	0.81990	-0.00367
ITEM037	-0.03332	0.02278	0.05559	0.80228	-0.02228
ITEM001	-0.08968	-0.00920	-0.01159	0.73106	0.00189
ITEM061	-0.06973	0.00926	0.00643	0.68103	-0.02209
ITEM038	-0.06393	0.07087	-0.00541	0.53365	-0.08246
ITEM083	0.08732	0.00832	-0.03882	0.34865	0.02123
ITEM077	-0.02247	0.06997	-0.02398	0.01489	0.76350
ITEM097	0.04599	0.03749	0.01514	-0.07239	0.71389
ITEM139	0.00215	0.06398	-0.06880	-0.09229	0.70360
ITEM118	-0.05851	0.11143	-0.07272	-0.06931	0.56177
ITEM054	0.00656	0.05603	-0.02984	-0.03272	0.55232
ITEM012	-0.01040	0.08183	-0.01685	0.12213	0.53413
ITEM087	0.13428	-0.01373	0.01387	-0.07160	-0.03037
ITEM064	0.13836	-0.05695	-0.00238	-0.04873	-0.02151
ITEM106	0.08031	0.00876	-0.00984	-0.09468	-0.03652
ITEM004	-0.08207	-0.06276	-0.05309	-0.01091	0.01729
ITEM041	0.07918	0.06190	0.05239	-0.03119	0.02600
ITEM022	-0.02143	0.14389	-0.06905	-0.06168	0.05496
ITEM128	0.23503	0.04537	0.03475	-0.05813	-0.05129
ITEM049	-0.04625	-0.06472	-0.02550	-0.05444	-0.07454
ITEM072	0.00253	0.00074	-0.01180	-0.06308	-0.09837
ITEM026	0.02694	-0.01281	-0.04848	-0.01176	-0.07391
ITEM008	-0.03437	-0.09647	-0.10775	0.03406	-0.11334
ITEM114	0.02042	-0.01682	-0.02343	-0.02554	0.00647
ITEM080	-0.01602	-0.00970	0.04670	0.02139	0.01781
ITEM058	-0.06104	-0.03643	0.10223	0.00651	0.00300
ITEM100	0.00928	-0.02621	0.00400	0.05369	0.06353
ITEM121	-0.07492	0.02371	-0.03088	0.08950	0.05474
ITEM081	0.00644	-0.06884	0.00814	-0.07056	0.04618
ITEM034	0.03773	-0.11227	-0.03539	-0.04242	-0.04555
ITEM015	0.02426	-0.06358	-0.03555	-0.01682	-0.03820
ITEM057	0.02398	-0.12241	0.00340	-0.06201	-0.05618
ITEM079	0.09109	0.00162	0.03292	-0.08042	0.01024
ITEM016	-0.11809	-0.05767	-0.08643	-0.00727	0.03087
ITEM137	-0.03791	-0.00986	0.04753	-0.09227	0.02744
ITEM115	0.13685	-0.01057	0.12347	-0.03346	-0.01338
ITEM093	0.14072	0.01508	0.16585	-0.00163	-0.03012

	FACTOR1	FACTOR2	FACTOR3	FACTOR4	FACTOR5
ITEM073	0.06855	-0.02039	-0.01656	-0.04378	0.01830
ITEM068	0.34055	-0.05129	0.19424	-0.06631	-0.00645
ITEM060	-0.02166	0.03262	0.01137	-0.01266	0.04509
ITEM082	0.02938	0.02182	-0.00459	0.00397	0.13105
ITEM059	-0.01162	0.05171	0.01690	-0.00422	-0.02064
ITEM102	-0.02097	0.06751	-0.04327	-0.06429	0.19286
ITEM018	-0.07701	-0.04038	-0.11072	-0.04016	-0.00378
ITEM123	0.10905	0.04031	0.07102	-0.04645	0.09975
ITEM117	0.21398	0.16493	0.20724	-0.07785	-0.01450
ITEM131	0.22795	0.08170	0.21005	-0.05344	0.02461
ITEM095	0.19822	0.00653	0.25942	-0.07595	-0.05923
ITEM105	0.13442	0.11530	0.02900	-0.04265	-0.03743
ITEM084	0.11544	0.08077	-0.04913	-0.02492	0.02969
ITEM103	-0.03583	0.01610	-0.00338	-0.08212	0.01461
ITEM125	-0.03104	0.05064	-0.05264	-0.06468	0.09143
ITEM062	0.04606	-0.09450	0.04405	-0.01689	-0.04787
ITEM071	0.16786	0.03434	0.02625	0.01829	0.01901
ITEM092	0.23652	0.07110	0.01292	-0.02549	-0.00578
ITEM007	0.05564	0.02820	-0.13498	0.03018	-0.05643
ITEM135	0.05052	-0.02909	0.06049	-0.06133	0.00836
ITEM113	0.10515	0.09908	0.02236	-0.06909	-0.02433
ITEM091	0.01045	0.06644	0.09689	0.10439	0.05004
ITEM021	0.05353	-0.00378	0.03112	-0.02677	-0.02816
ITEM039	0.15879	0.04348	0.07376	0.05420	0.04183
ITEM063	0.28508	-0.01725	0.05862	0.01530	-0.03287
ITEM040	0.28332	-0.08929	0.09035	0.02981	-0.00232
ITEM085	0.26947	0.01511	-0.01561	-0.03176	-0.02548
ITEM027	0.06032	-0.06574	-0.01300	-0.06551	-0.03625
ITEM009	0.04335	-0.03051	-0.05948	-0.01090	-0.05459
ITEM050	-0.03953	-0.05029	0.01777	-0.06458	0.01757
ITEM023	-0.00908	0.03037	-0.05497	-0.05439	-0.02586
ITEM005	-0.05294	0.03544	0.02955	0.07714	0.04275
ITEM069	-0.00898	-0.01526	0.10344	-0.00102	-0.02853
ITEM030	-0.03783	0.11907	-0.07584	0.09941	-0.02602
ITEM076	0.00879	0.13724	-0.06022	0.08708	0.12442

	FACTOR6	FACTOR7	FACTOR8	FACTOR9	FACTOR10
ITEM090	0.07513	0.05258	0.02909	0.02291	0.11983
ITEM044	-0.02452	0.03170	-0.04191	0.03948	-0.02650
ITEM089	0.04251	0.06350	-0.00691	0.05350	-0.05221
ITEM045	-0.12722	-0.02672	-0.08681	0.02183	-0.02800
ITEM065	0.17723	-0.00766	0.04971	0.01647	0.07884
ITEM043	-0.01699	-0.06297	-0.06324	0.09410	-0.06547
ITEM042	0.08328	0.03319	-0.05012	-0.00478	0.16657
ITEM088	0.20366	-0.01855	0.02402	-0.03779	0.09804
ITEM108	0.03303	-0.05151	-0.05048	0.01104	0.01703
ITEM046	0.00947	-0.03142	-0.04812	-0.11166	0.11073
ITEM109	0.12144	-0.04252	0.06083	-0.04197	0.10087
ITEM119	0.00521	-0.02040	0.05461	-0.11156	0.00407
ITEM056	0.01223	-0.01929	0.02698	-0.07784	0.00135
ITEM033	-0.00203	-0.09031	-0.10156	-0.01859	-0.01024
ITEM140	0.05135	0.05440	-0.02397	-0.04373	0.03677
ITEM098	0.00866	-0.02542	0.02266	-0.07362	-0.03056
ITEM120	0.05038	-0.03348	0.04014	-0.01996	0.00675
ITEM053	-0.01138	-0.07584	-0.10578	-0.01250	-0.00435
ITEM011	-0.06651	-0.15881	-0.13918	-0.00582	-0.08847
ITEM055	0.03184	0.10546	-0.00747	-0.00513	0.04500
ITEM074	0.04172	-0.01782	0.08394	0.00425	0.12282
ITEM052	-0.05221	-0.10243	-0.04669	-0.02287	-0.00930
ITEM051	-0.04676	0.04050	0.01407	0.04997	-0.05925
ITEM094	0.01222	0.00168	0.09288	-0.04297	0.14677
ITEM075	0.05695	0.00088	0.01585	-0.07109	0.10986
ITEM029	-0.00114	-0.09554	-0.12183	-0.02188	-0.10738
ITEM116	-0.08259	-0.12124	-0.01507	-0.02284	0.17561
ITEM138	-0.00779	-0.09403	0.08415	0.00382	0.12717
ITEM019	-0.00363	-0.03427	0.03649	-0.04252	-0.00291
ITEM037	-0.05252	-0.03355	0.03355	-0.05485	0.01993
ITEM001	-0.05607	0.00192	0.03969	-0.04795	-0.09703
ITEM061	-0.10432	-0.05671	0.02109	-0.02519	-0.05711
ITEM038	-0.07221	0.00169	-0.05299	0.02476	0.01060
ITEM083	-0.08680	-0.00961	0.10037	-0.20406	-0.13079
ITEM077	0.00129	-0.09233	0.04601	-0.07895	0.04741
ITEM097	0.01594	-0.03522	0.09241	-0.04972	-0.01323
ITEM139	0.02299	-0.07400	-0.00320	0.05113	0.07886
ITEM118	-0.02780	-0.06643	0.00147	-0.00721	0.07723
ITEM054	0.01230	-0.06146	0.03032	0.00158	-0.05722

	FACTOR6	FACTOR7	FACTOR8	FACTOR9	FACTOR10
ITEM012	-0.04564	-0.03592	-0.00312	-0.03566	-0.12512
ITEM087	0.72468	0.04697	0.00493	-0.03663	0.03413
ITEM064	0.65759	0.02597	0.00433	-0.02130	-0.01576
ITEM106	0.62637	0.07134	0.05401	-0.06665	0.03466
ITEM004	0.53799	-0.01490	-0.02211	0.01198	-0.04102
ITEM041	0.51336	-0.06638	-0.04678	0.08749	0.05997
ITEM022	0.48730	-0.00972	-0.00313	0.00762	0.04055
ITEM128	0.48667	-0.09256	-0.05296	0.00126	-0.06492
ITEM049	-0.00687	0.76184	-0.04822	0.00882	-0.02056
ITEM072	0.02489	0.69637	-0.05862	0.00956	0.07426
ITEM026	-0.02672	0.66310	-0.10192	0.01462	-0.09282
ITEM008	-0.07695	0.58966	-0.11333	0.00863	-0.20404
ITEM114	0.04896	0.56453	-0.02570	-0.01104	-0.02929
ITEM080	-0.00157	-0.01788	0.72097	0.08588	-0.02865
ITEM058	-0.02057	-0.02940	0.65040	0.17280	-0.09884
ITEM100	0.00243	-0.08721	0.64978	-0.00306	-0.04072
ITEM121	-0.02254	-0.18975	0.59427	-0.02544	0.00903
ITEM081	0.02733	-0.06339	0.38517	0.37742	0.01478
ITEM034	-0.03415	0.05994	-0.04035	0.76103	-0.03756
ITEM015	0.04660	0.00303	0.03714	0.72110	0.00639
ITEM057	-0.01092	0.01406	0.17541	0.71653	-0.13192
ITEM079	-0.00715	-0.01106	0.33868	0.47274	0.02674
ITEM016	-0.07861	-0.19080	0.36118	0.36325	-0.14534
ITEM137	0.02501	-0.14584	-0.01135	-0.04493	0.70127
ITEM115	-0.01675	-0.04071	-0.13224	-0.06606	0.67542
ITEM093	-0.00477	-0.01725	-0.09516	-0.09281	0.65720
ITEM073	0.09552	-0.03987	0.03670	0.06623	0.52773
ITEM068	0.01034	-0.06041	-0.04576	-0.08994	0.35228
ITEM060	0.07313	0.00279	-0.03072	0.03829	-0.06773
ITEM082	0.02972	-0.02126	0.15242	-0.01637	0.06432
ITEM059	0.00829	-0.05460	0.06354	-0.02201	0.00856
ITEM102	0.05911	-0.04164	0.02966	-0.00062	0.00290
ITEM018	-0.05191	0.03474	-0.05674	0.05933	-0.11786
ITEM123	0.05378	0.00222	-0.04014	0.06385	-0.06170
ITEM117	0.02490	0.00131	-0.00642	-0.06710	0.13590
ITEM131	0.03158	-0.02365	-0.03582	0.05548	0.08979
ITEM095	0.03623	-0.04473	-0.00349	-0.09222	0.14158
ITEM105	0.06500	0.05639	0.03699	-0.05976	0.13463
ITEM084	-0.03079	0.02230	0.00496	-0.06976	-0.07590
ITEM103	0.05257	0.02469	-0.03153	0.03053	0.19115
ITEM125	-0.06660	-0.01976	-0.04983	-0.05723	0.03907
ITEM062	0.08331	0.06853	0.03978	0.01391	0.07295
ITEM071	0.03413	0.04665	-0.03348	-0.09258	0.11737
ITEM092	-0.00891	0.02008	0.07110	-0.09226	0.14775
ITEM007	-0.00309	0.08046	-0.08417	-0.00466	-0.16784
ITEM135	0.04173	-0.06614	-0.02367	0.09057	0.05172
ITEM113	-0.03404	0.08970	-0.04214	0.01587	0.09595
ITEM091	-0.11746	-0.06496	0.04735	-0.20235	0.10703
ITEM021	0.13698	0.04676	-0.03712	-0.03968	-0.01906
ITEM039	0.16497	0.07744	0.02735	-0.03777	0.03078
ITEM063	0.18293	0.00255	-0.02279	-0.01350	-0.07651
ITEM040	0.13194	-0.04672	-0.07252	0.06832	0.03957
ITEM085	0.09254	-0.05522	-0.04856	-0.00149	-0.09604
ITEM027	0.00150	0.01629	-0.03109	0.01452	0.12653
ITEM009	0.01378	0.02989	-0.01384	0.03726	0.09644
ITEM050	-0.04275	0.05896	-0.03939	-0.07164	0.05606
ITEM023	0.06102	-0.00680	-0.00408	0.07143	-0.02483
ITEM005	0.01326	-0.02043	0.03512	-0.02718	-0.04865
ITEM069	0.03569	0.07086	0.07114	0.15232	-0.03364
ITEM030	0.06062	0.04428	0.16140	-0.04992	-0.01393
ITEM076	-0.00905	0.02990	0.13047	0.01287	0.01381
ITEM124	-0.06661	-0.08552	0.18250	0.00023	-0.00580

	FACTOR11	FACTOR12	FACTOR13	FACTOR14	FACTOR15
ITEM090	-0.00055	0.10059	0.07177	0.21718	-0.00847
ITEM044	-0.07273	0.09960	-0.00636	-0.00919	0.24978
ITEM089	0.03734	0.01747	0.05394	0.13198	0.10261
ITEM045	-0.04752	0.10078	0.00988	-0.02131	0.31212

	FACTOR11	FACTOR12	FACTOR13	FACTOR14	FACTOR15
ITEM065	0.01495	-0.03645	0.03470	0.06171	0.01328
ITEM043	-0.07653	0.01459	-0.03021	0.00885	0.19216
ITEM042	-0.00579	0.12149	-0.04006	0.00062	-0.08784
ITEM088	0.03678	0.08187	0.04939	0.05669	-0.07960
ITEM108	-0.00248	0.12045	-0.00354	0.15296	0.18589
ITEM046	-0.03081	0.17153	-0.05877	0.22022	-0.03025
ITEM109	0.05474	0.26738	-0.06620	0.12240	0.18355
ITEM119	-0.03298	0.11685	-0.03553	-0.03276	-0.01674
ITEM056	0.00472	0.01273	0.01041	-0.06713	0.07789
ITEM033	0.00055	-0.05264	-0.01114	0.02447	-0.00997
ITEM140	0.07854	0.15683	0.03546	0.08543	-0.01919
ITEM098	0.09575	0.06543	-0.00173	0.03977	-0.02901
ITEM120	-0.04945	0.26672	0.03233	-0.00500	-0.13113
ITEM053	0.11279	-0.15212	0.02105	0.08595	0.02938
ITEM011	-0.03141	0.01366	0.00379	0.08284	-0.01368
ITEM055	0.05483	-0.18338	0.03883	0.15524	0.13070
ITEM074	0.01882	0.00516	-0.03677	0.02517	0.03039
ITEM052	-0.05878	0.09643	-0.04674	0.03690	0.00887
ITEM051	-0.02073	-0.07009	-0.02756	-0.01324	0.12243
ITEM094	0.00174	0.15466	-0.00160	0.04107	0.10870
ITEM075	0.03625	0.11353	0.10643	-0.04193	-0.15780
ITEM029	-0.11201	0.28697	0.03689	-0.08521	-0.03596
ITEM116	-0.07783	0.23601	-0.08722	0.08089	0.03084
ITEM138	0.04052	0.15136	-0.00030	0.02695	0.14039
ITEM019	-0.04007	-0.00600	-0.01149	0.02582	0.01849
ITEM037	-0.05584	-0.02963	0.01657	0.01382	0.01492
ITEM001	0.00120	0.04202	-0.02942	-0.02396	-0.01931
ITEM061	-0.05156	-0.09189	-0.05800	-0.00804	-0.06481
ITEM038	0.02384	-0.08376	-0.07260	-0.06312	0.12305
ITEM083	-0.01842	-0.05492	-0.14141	-0.12872	-0.13409
ITEM077	0.05319	-0.07135	0.03196	-0.03640	-0.03708
ITEM097	0.09396	0.05167	0.01160	0.01096	-0.03337
ITEM139	0.11084	0.06955	0.00826	0.05555	0.04142
ITEM118	0.04329	0.06787	0.01383	0.02924	0.03251
ITEM054	0.13712	-0.18638	0.00261	-0.05567	0.03804
ITEM012	-0.03315	0.00160	0.02011	-0.01840	-0.03982
ITEM087	0.06758	-0.00399	0.05024	0.03125	0.07028
ITEM064	0.04544	-0.12321	0.07006	-0.00758	0.07705
ITEM106	0.08292	0.14945	0.06590	0.04284	0.14974
ITEM004	-0.01875	0.12196	-0.06797	-0.05343	0.09667
ITEM041	-0.00186	-0.04868	-0.03664	-0.04253	0.02067
ITEM022	-0.01841	0.03572	-0.07989	0.00911	0.07786
ITEM128	0.03506	0.02874	0.12608	0.10012	0.05656
ITEM049	-0.02839	-0.03512	0.05764	-0.10283	0.03570
ITEM072	0.07030	-0.12348	0.13260	-0.06769	0.00083
ITEM026	-0.05662	0.03456	-0.02831	0.09096	-0.00198
ITEM008	-0.06736	0.05233	-0.07796	0.12406	0.02524
ITEM114	-0.02349	0.04499	-0.00093	0.06641	0.01900
ITEM080	0.02841	-0.03321	-0.01051	-0.11804	0.01846
ITEM058	-0.01342	-0.03565	-0.02696	-0.10880	-0.05558
ITEM100	0.12694	0.04462	-0.00035	0.07045	-0.03633
ITEM121	-0.06410	0.06775	0.00330	0.13468	-0.00494
ITEM081	0.21901	-0.17236	0.03332	-0.07926	-0.04575
ITEM034	0.00145	-0.02772	-0.01153	-0.03876	0.00082
ITEM015	-0.03648	0.09488	-0.07933	0.03763	-0.00068
ITEM057	0.04712	-0.10885	-0.00813	-0.06966	-0.01763
ITEM079	0.07108	-0.14445	0.03056	-0.09687	-0.00189
ITEM016	-0.05007	0.09921	-0.07068	0.13552	-0.05636
ITEM137	-0.03570	0.04390	0.10307	0.06025	0.02559
ITEM115	-0.08760	0.24200	0.02266	0.04894	-0.04841
ITEM093	-0.03275	0.11246	0.09094	0.13009	0.00559
ITEM073	0.01190	-0.03675	0.00118	0.15762	-0.00871
ITEM068	-0.05405	0.26627	0.02763	0.01408	-0.05337
ITEM060	0.66063	-0.08307	-0.01971	0.05259	0.06476
ITEM082	0.61794	-0.08661	-0.02322	-0.03934	0.00092
ITEM059	0.58995	-0.02263	0.00477	-0.10237	0.04478
ITEM102	0.58371	0.13029	-0.01497	0.07151	-0.01361
ITEM018	0.54571	0.02127	-0.09283	0.00921	0.09392
ITEM123	0.40519	0.25232	-0.01953	0.04126	-0.02507
ITEM117	0.02009	0.54388	0.05825	0.04152	-0.04037
ITEM131	-0.03447	0.53659	-0.01680	0.13379	0.02662
ITEM095	-0.04457	0.53167	0.14445	0.01287	-0.07336
ITEM105	0.05796	0.49543	0.13521	-0.02150	0.18637
ITEM084	-0.04304	0.03239	0.74360	-0.03321	-0.03362
ITEM103	-0.05763	0.13917	0.73206	0.03962	0.06754

	FACTOR11	FACTOR12	FACTOR13	FACTOR14	FACTOR15
ITEM125	-0.09506	0.04376	0.69439	0.03340	-0.00521
ITEM062	0.04005	-0.02489	0.63424	0.03932	0.05841
ITEM071	0.02467	-0.05094	0.05245	0.58052	-0.10590
ITEM092	0.07189	0.00699	0.04006	0.55090	0.03700
ITEM007	-0.06197	0.13348	-0.05080	0.54613	-0.02494
ITEM135	0.01537	-0.04713	0.08491	0.54568	0.02200
ITEM113	-0.05099	0.13699	-0.07991	0.46896	0.04213
ITEM091	-0.07170	0.07026	-0.01070	0.41287	-0.06020
ITEM021	0.04274	0.06488	-0.00216	-0.08400	0.67496
ITEM039	0.06172	-0.00958	-0.00516	-0.03142	0.66247
ITEM063	0.11993	0.01218	0.11932	0.07328	0.46853
ITEM040	0.00125	-0.05269	0.00926	0.04465	0.45453
ITEM085	0.18599	-0.00784	0.10729	0.01243	0.31435
ITEM027	0.05659	0.04095	-0.00063	0.09673	0.03285
ITEM009	0.03070	0.03473	0.02362	0.10544	0.04634
ITEM050	0.01359	-0.10770	-0.02396	0.02685	-0.00122
ITEM023	0.00082	-0.02731	-0.06360	-0.00096	0.04455
ITEM005	-0.02967	0.08572	-0.08452	0.00710	-0.04938
ITEM069	0.09770	-0.08012	0.02489	-0.00641	-0.03894
ITEM030	0.10821	-0.02239	-0.03743	-0.01157	0.05866
ITEM076	0.19776	-0.16023	-0.01510	-0.05525	-0.01760
ITEM124	-0.10834	0.08725	0.05185	0.19240	-0.11788

	FACTOR16	FACTOR17	FACTOR18	FACTOR19
ITEM090	-0.02532	-0.04333	0.04895	-0.13096
ITEM044	0.02454	0.08192	-0.06460	-0.02020
ITEM089	0.00117	-0.05141	0.15467	-0.09044
ITEM045	-0.01064	0.08161	-0.01993	0.00136
ITEM065	0.00282	-0.09121	-0.02737	0.07652
ITEM043	0.03942	0.04577	0.03838	0.17953
ITEM042	0.07431	-0.05874	-0.21597	0.12268
ITEM088	0.00977	-0.02302	0.04675	-0.08238
ITEM108	-0.03662	-0.01414	0.27698	0.01224
ITEM046	0.00237	-0.00549	-0.25287	-0.01768
ITEM109	-0.04715	0.01956	0.20436	-0.19891
ITEM119	-0.00366	0.02027	0.15370	-0.03392
ITEM056	-0.02957	-0.00565	-0.04495	-0.02477
ITEM033	-0.08433	0.11216	-0.23309	0.12862
ITEM140	-0.02664	-0.11694	0.17331	0.04088
ITEM098	0.01424	0.05700	0.04534	-0.09010
ITEM120	0.08071	-0.07097	0.14797	0.00288
ITEM053	-0.10615	0.03694	-0.10898	0.26423
ITEM011	-0.05705	0.08757	-0.29596	0.08800
ITEM055	-0.05637	0.02276	-0.02415	0.19262
ITEM074	-0.08342	0.04258	-0.04985	-0.13096
ITEM052	0.10677	0.01541	-0.04425	0.18440
ITEM051	0.06368	-0.09336	0.01136	-0.09457
ITEM094	-0.15584	0.02447	0.00368	-0.16068
ITEM075	0.06559	-0.00228	0.02760	0.02426
ITEM029	0.07587	0.09435	-0.11444	0.25368
ITEM116	-0.16375	0.12070	0.15232	0.06379
ITEM138	-0.07362	0.04586	0.15670	-0.19055
ITEM019	-0.05512	0.02222	-0.08537	0.08587
ITEM037	-0.08033	-0.00214	-0.05860	0.05537
ITEM001	-0.01106	-0.03047	-0.08089	0.04766
ITEM061	-0.07234	0.07888	0.05314	0.07498
ITEM038	0.07675	-0.05780	0.06366	-0.10029
ITEM083	-0.07391	0.19958	0.20477	0.07798
ITEM077	-0.07484	0.07804	-0.07516	-0.00425
ITEM097	-0.02004	-0.01387	-0.04340	-0.15914
ITEM139	0.01912	-0.00653	0.18373	-0.00177
ITEM118	0.04239	0.06802	0.31049	0.10080
ITEM054	-0.02832	-0.14995	-0.01863	0.10079
ITEM012	-0.00346	0.02920	-0.19833	0.12468
ITEM087	-0.03004	-0.05283	0.08871	-0.06847
ITEM064	-0.00907	-0.09116	0.06830	-0.00167
ITEM106	-0.03232	-0.08501	0.16259	-0.01817
ITEM004	0.04594	0.04326	-0.07408	0.04283
ITEM041	0.00392	0.13951	-0.11838	-0.01147
ITEM022	-0.01735	0.15009	-0.14285	0.04609
ITEM128	0.02969	-0.01849	0.34184	0.06397
ITEM049	-0.00729	-0.09763	-0.05064	-0.00891

	FACTOR16	FACTOR17	FACTOR18	FACTOR19
ITEM072	0.04357	0.01173	0.05563	-0.02517
ITEM026	0.04063	0.04531	-0.14141	0.00224
ITEM008	-0.02166	0.04816	-0.09034	0.00618
ITEM114	0.04022	0.01341	0.17596	0.05852
ITEM080	-0.01031	0.03001	-0.01247	0.01317
ITEM058	-0.00628	0.00443	-0.06531	-0.04399
ITEM100	-0.05771	0.04839	0.02879	0.10757
ITEM121	-0.00639	0.00925	0.04495	0.23207
ITEM081	-0.01339	0.03210	0.12154	-0.04967
ITEM034	0.02156	0.02985	0.00894	-0.00079
ITEM015	-0.00424	0.04711	-0.10536	-0.01775
ITEM057	-0.03573	0.03499	0.06400	0.02400
ITEM079	-0.00619	0.06854	0.14185	-0.07856
ITEM016	0.00800	-0.01619	-0.19403	0.12041
ITEM137	0.18207	-0.06896	0.01699	0.00229
ITEM115	0.00317	-0.01397	0.01269	0.01451
ITEM093	-0.01354	0.01934	-0.01740	0.01306
ITEM073	0.34953	-0.06080	0.00382	-0.07803
ITEM068	0.13116	0.01596	-0.16207	0.00692
ITEM060	0.00459	-0.01955	-0.00369	0.01285
ITEM082	-0.04191	0.04644	0.01117	0.08917
ITEM059	0.10835	0.01802	-0.01998	-0.10404
ITEM102	0.00560	-0.01354	0.07692	0.03259
ITEM018	0.00736	0.02185	-0.05540	0.15231
ITEM123	0.04563	-0.07873	0.32621	0.16875
ITEM117	-0.01184	-0.00236	0.04275	0.02845
ITEM131	-0.03600	0.03819	0.11245	-0.11223
ITEM095	0.02952	-0.04609	-0.19510	0.02678
ITEM105	-0.06866	0.00078	0.01723	-0.15493
ITEM084	-0.06175	0.01646	0.01215	0.03153
ITEM103	0.01464	-0.07776	-0.01985	-0.04111
ITEM125	0.01012	-0.02971	0.14921	0.11018
ITEM062	0.04022	-0.07599	-0.10092	-0.14644
ITEM071	0.10194	0.04724	-0.05975	-0.02716
ITEM092	0.04310	-0.04656	-0.03959	-0.06221
ITEM007	0.12617	0.09201	-0.21969	0.09971
ITEM135	0.06183	-0.01828	0.13423	-0.06008
ITEM113	0.02070	-0.07004	0.16805	0.05583
ITEM091	-0.09299	0.38159	-0.08525	0.05890
ITEM021	0.01146	0.03171	-0.03532	0.04677
ITEM039	0.01804	-0.04921	0.01080	0.01448
ITEM063	0.03157	-0.10287	0.06389	-0.14040
ITEM040	0.05928	-0.04056	0.03109	0.08115
ITEM027	0.75349	-0.00408	-0.04247	-0.01768
ITEM009	0.73503	0.01173	-0.06796	-0.07487
ITEM050	0.65578	-0.03634	0.11085	-0.01205
ITEM023	0.01574	0.66818	-0.00770	0.02246
ITEM005	0.01140	0.66638	-0.11116	-0.02602
ITEM069	-0.05613	0.62049	0.15300	0.07959
ITEM030	-0.06281	0.05993	-0.05174	0.58223
ITEM076	-0.09629	0.04463	0.07952	0.53559
ITEM124	-0.00325	-0.03848	0.17762	0.31377

Variance explained by each factor

FACTOR1	FACTOR2	FACTOR3	FACTOR4	FACTOR5	FACTOR6	FACTOR7	FACTOR8	FACTOR9	FACTOR10
3.961424	3.401198	3.118868	3.097631	2.854452	2.766337	2.538690	2.513977	2.472766	2.461073

FACTOR11	FACTOR12	FACTOR13	FACTOR14	FACTOR15	FACTOR16	FACTOR17	FACTOR18	FACTOR19
2.399937	2.290576	2.276704	2.214324	2.058544	1.988466	1.800575	1.666343	1.641151

Final Communality Estimates: Total = 47.523034

Rotation Method: Varimax

ITEM001	ITEM004	ITEM005	ITEM007	ITEM008	ITEM009	ITEM011	ITEM012	ITEM015	ITEM016	ITEM018
0.572592	0.349225	0.492724	0.473670	0.484649	0.588017	0.435989	0.386786	0.562922	0.443222	0.388558

ITEM019	ITEM021	ITEM022	ITEM023	ITEM026	ITEM027	ITEM029	ITEM030	ITEM033	ITEM034	ITEM037
0.703229	0.502865	0.331524	0.471207	0.505663	0.616375	0.483674	0.432681	0.487793	0.609964	0.674923

ITEM038	ITEM039	ITEM040	ITEM041	ITEM042	ITEM043	ITEM044	ITEM045	ITEM046	ITEM049	ITEM050
0.364800	0.520427	0.352552	0.335455	0.392642	0.387670	0.466114	0.468126	0.346226	0.628213	0.480893

ITEM051	ITEM052	ITEM053	ITEM054	ITEM055	ITEM056	ITEM057	ITEM058	ITEM059	ITEM060	ITEM061
0.457647	0.509644	0.450572	0.410214	0.361063	0.517078	0.610941	0.502862	0.395535	0.467469	0.526354

ITEM062	ITEM063	ITEM064	ITEM065	ITEM068	ITEM069	ITEM071	ITEM072	ITEM073	ITEM074	ITEM075
0.482762	0.415383	0.500253	0.375103	0.420216	0.483554	0.428655	0.556515	0.460970	0.535163	0.457787

ITEM076	ITEM077	ITEM079	ITEM080	ITEM081	ITEM082	ITEM083	ITEM084	ITEM085	ITEM087	ITEM088
0.436778	0.637258	0.422299	0.549249	0.413356	0.450956	0.358867	0.599171	0.353614	0.583636	0.368140

ITEM089	ITEM090	ITEM091	ITEM092	ITEM093	ITEM094	ITEM095	ITEM097	ITEM098	ITEM100	ITEM102
0.400971	0.493059	0.449254	0.418867	0.538324	0.515928	0.500640	0.571980	0.378390	0.481660	0.424730

ITEM103	ITEM105	ITEM106	ITEM108	ITEM109	ITEM113	ITEM114	ITEM115	ITEM116	ITEM117	ITEM118
0.623428	0.397193	0.515759	0.374240	0.390359	0.333319	0.368204	0.587430	0.406869	0.451577	0.474292

ITEM119	ITEM120	ITEM121	ITEM123	ITEM124	ITEM125	ITEM128	ITEM131	ITEM135	ITEM137	ITEM138
0.581718	0.443609	0.491413	0.416785	0.403670	0.560606	0.470293	0.456204	0.362144	0.585195	0.399851

ITEM139	ITEM140
0.583412	0.431228
